# Large Language Models in Critical Care Medicine: Scoping Review

**DOI:** 10.2196/76326

**Published:** 2025-11-24

**Authors:** Tongyue Shi, Jun Ma, Zihan Yu, Haowei Xu, Rongxin Yang, Minqi Xiong, Meirong Xiao, Yilin Li, Huiying Zhao, Guilan Kong

**Affiliations:** 1 National Institute of Health Data Science Peking University Beijing China; 2 Institute for Artificial Intelligence Peking University Beijing China; 3 Institute of Medical Technology Peking University Health Science Center Beijing China; 4 Advanced Institute of Information Technology Peking University Hangzhou China; 5 Peking University Third Hospital Beijing China; 6 Department of Computer Science University of Liverpool Liverpool United Kingdom; 7 Johns Hopkins University School of Medicine Baltimore, MD United States; 8 Fielding School of Public Health University of California, Los Angeles Los Angeles, CA United States; 9 Department of Critical Care Medicine Peking University People's Hospital Beijing China

**Keywords:** artificial intelligence, large language model, ChatGPT, critical care, intensive care, clinical decision support, intensive care unit

## Abstract

**Background:**

With the rapid development of artificial intelligence, large language models (LLMs) have shown strong capabilities in natural language understanding, reasoning, and generation, attracting much research interest in applying LLMs to health and medicine. Critical care medicine (CCM) provides diagnosis and treatment for patients with critical illness who often require intensive monitoring and interventions in intensive care units (ICUs). Whether LLMs can be applied to CCM, and whether they can operate as ICU experts in assisting clinical decision-making rather than “stochastic parrots,” remains uncertain.

**Objective:**

This scoping review aims to provide a panoramic portrait of the application of LLMs in CCM, identifying the advantages, challenges, and future potential of LLMs in this field.

**Methods:**

This study was conducted in accordance with the PRISMA-ScR (Preferred Reporting Items for Systematic Reviews and Meta-Analyses extension for Scoping Reviews) guidelines. Literature was searched across 7 databases, including PubMed, Embase, Scopus, Web of Science, CINAHL, IEEE Xplore, and ACM Digital Library, from the first available paper to August 22, 2025.

**Results:**

From an initial 2342 retrieved papers, 41 were selected for final review. LLMs played an important role in CCM through the following 3 main channels: clinical decision support, medical documentation and reporting, and medical education and doctor-patient communication. Compared to traditional artificial intelligence models, LLMs have advantages in handling unstructured data and do not require manual feature engineering. Meanwhile, applying LLMs to CCM has faced challenges, including hallucinations and poor interpretability, sensitivity to prompts, bias and alignment challenges, and privacy and ethical issues.

**Conclusions:**

Although LLMs are not yet ICU experts, they have the potential to become valuable tools in CCM, helping to improve patient outcomes and optimize health care delivery. Future research should enhance model reliability and interpretability, improve model training and deployment scalability, integrate up-to-date medical knowledge, and strengthen privacy and ethical guidelines, paving the way for LLMs to fully realize their impact in critical care.

**Trial Registration:**

OSF Registries yn328; https://osf.io/yn328/

## Introduction

Critical care medicine (CCM), or intensive care medicine, is an essential field dedicated to managing severely ill patients, emphasizing rapid and life-critical decision-making and interventions. CCM deals with patients who have severe conditions and injuries such as acute kidney injury (AKI), sepsis, and acute respiratory distress syndrome (ARDS), potentially leading to a deteriorative state in the intensive care units (ICUs) [1-4]. The incidence of AKI in the ICU could reach over 50% worldwide [5]. Among those who received renal replacement therapy, most of whom were with critical illness in the ICU, the mortality rate was approximately 50% [6,7]. The prevalence of sepsis is around 30% during ICU stay [8]. Sepsis accounted for approximately 11 million deaths, making up about 20% of all global deaths [9]. Recent multicenter epidemiological work shows that the incidence of ARDS in the ICU was between 7.1% and 19% with hospital mortality of 32%-55% [10]. While in resource-limited settings, the ICU mortality of ARDS could be as high as 50% due to the disparities in health care services [11]. Therefore, the special environment of the ICU has imposed higher professional requirements on medical staff. Physicians and nurses in ICUs must manage large amounts of patient data while maintaining high efficiency under high pressure [11,12]. The dynamic and severe nature of critical care demands intelligent decision-support tools to help physicians improve diagnostic accuracy, optimize therapeutic strategies, and facilitate timely clinical decision-making.

Artificial intelligence (AI) technologies, especially generative artificial intelligence (GenAI) models, have developed rapidly in recent years [13,14]. The advent of large language models (LLMs), such as those based on the Transformer architecture [15] and pretrained on extensive text corpora, has marked a substantial advancement in natural language processing (NLP). With billions of parameters, these LLMs have demonstrated remarkable capabilities in understanding and generating human-like text [16]. LLMs have been implemented in various contexts, including answering questions, summarizing texts, and engaging in open-domain conversations [17]. Compared to human practitioners, LLMs have been perceived as more understanding and efficient [18]. Among these LLMs, OpenAI’s ChatGPT [16] has become a focal point since its launch in November 2022. OpenAI then introduced upgraded versions of ChatGPT, offering enhanced multimodal capabilities to handle diverse inputs such as text, images, code, and table files. LLMs have revolutionized different fields, including health and medicine [13,14]. A more detailed description of the evolution and applications of LLMs in health and medicine is provided in Note S1 in Multimedia Appendix 1 [19-59].

In the field of CCM, the emergence of LLMs demonstrates its unique potential. Similar to the application of LLMs in informing patients with cancer of diagnosis, treatment methods, and side effects, LLMs in CCM can help make life-or-death decisions after fusing large volumes of patient data in a short time [60]. Physicians in CCM face enormous workloads and pressure, involving LLMs in different clinical decision-making scenarios in CCM will help reduce the workload of physicians and improve health care quality. However, LLMs face challenges when applied in CCM, such as uncertain accuracy and coherence, recency bias, hallucinations, poor interpretability, and ethical issues [61]. Among them, hallucinations are one of the biggest drawbacks of LLMs, which make them act like stochastic parrots [62].

This study aims to review the applications of LLMs in CCM, identifying the advantages, challenges, and future potential of LLMs in this field. Three key research questions were designed to be answered by this review. (1) What is the current status of LLM applications within the critical care setting? (2) What are the recognized advantages and challenges of using LLMs in CCM? (3) What research directions should be taken in the future to promote the application of LLMs in CCM? By addressing the above 3 questions, this review endeavors to provide a clear portrait of and identify the research gaps in the applications of LLMs in CCM, discerning whether they are just stochastic parrots that may mimic human responses based on probability calculation or emerging ICU experts capable of providing timely and highly personalized diagnosis and treatment recommendations. Through this comprehensive review, we aim to outline a roadmap for future research and implementation of LLMs in CCM that could enable them to transform critical care effectively.

## Methods

### Study Design

This scoping review followed the PRISMA-ScR (Preferred Reporting Items for Systematic Reviews and Meta-Analyses extension for Scoping Reviews) guidelines [63], and the protocol was registered in the Open Science Framework. We have included a checklist of the PRISMA-ScR guidelines in Table S1 in Multimedia Appendix 1.

### Literature Search Strategy

We conducted a literature search across 7 databases, including PubMed, Embase, Scopus, Web of Science, CINAHL, IEEE Xplore, and ACM Digital Library, from the earliest available paper until August 22, 2025. Keywords related to LLMs included “large language model,” “LLM,” “generative pre-trained transformer,” “GPT,” “generative artificial intelligence,” and “generative AI.” For CCM, the keywords included “critical care,” “intensive care units,” “critical illness,” “intensive care,” and “ICU.” All these terms were combined using the “OR” and “AND” logical operators to ensure the retrieval of literature that addresses both research areas. The detailed search terms for each database are provided in Table S2 in Multimedia Appendix 1.

### Study Selection

The study selection in this scoping review was conducted to ensure comprehensive coverage and relevance of the included literature. In the first phase of the study selection, literature was included based on the following criteria: (1) focusing on LLMs in CCM, including studies that explicitly used or commented on LLMs relevant to the field of CCM, and (2) original research papers from peer-reviewed journals and conferences, perspectives, and letters. Studies were excluded from the review if they met any of the following conditions: (1) irrelevant to LLMs or CCM, including studies that did not focus on applying LLMs within the realm of CCM; (2) conference abstracts, preprint papers, books, patents, editorials, and review papers; and (3) non-English literature. The process for selecting sources of evidence is provided in Note S2 in Multimedia Appendix 1.

### Keyword Co-Occurrence Network Analysis

Keyword co-occurrence network analysis [64] is a bibliometric method to explore the relationships between keywords in academic papers. It involves constructing a network where nodes represent keywords and edges represent the co-occurrence of these keywords within the studied documents. It helps to identify the main research themes, trends, and potential research gaps by analyzing the frequency and patterns of keyword co-occurrences. This study used the VOSviewer (version 1.6.20) software to construct a bibliometric network using the visualization of similarity method [65,66]. The software automatically extracts keywords from a publication’s title, abstract, or author-supplied keyword list. The frequency of co-occurrences of 2 keywords is determined by the number of publications in which both keywords appear together in the title, abstract, or keyword list. The visualization of similarity method starts by calculating the similarity between the keywords of publications based on their co-occurrence. Finally, a matrix is constructed to arrange keywords spatially according to their similarities, and it is the basis for multivariate statistical and network analysis.

### Risk of Bias and Applicability Assessment

We critically appraised all included studies using the PROBAST-AI (Prediction Model Risk of Bias Assessment Tool-Artificial Intelligence) [67], rating Risk of Bias (RoB) across 4 domains (participants, predictors, outcome, and analysis) and applicability across 3 domains (participants, predictors, and outcome) on a 3-level scale (low, high, and unclear). Full evaluation criteria and rules are provided in Note S3 in Multimedia Appendix 1.

## Results

### Literature Search Results

**Figure 1:**
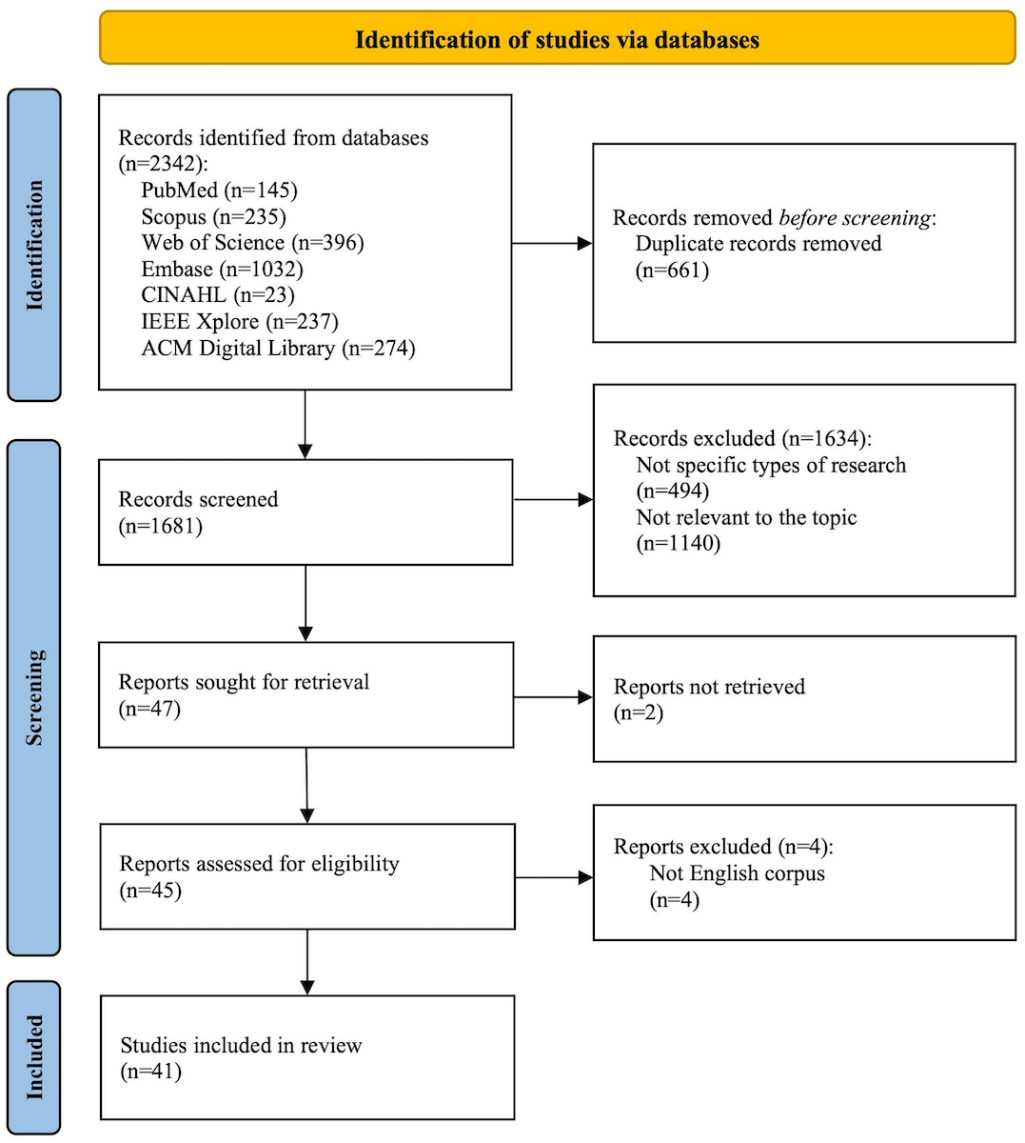
The PRISMA flowchart for study selection and quality assessment. PRISMA: Preferred Reporting Items for Systematic Reviews and Meta-Analyses.

This scoping review covered publications in the 7 databases to August 22, 2025, and retrieved 2342 papers initially. The flowchart of the study selection process is presented in Figure 1.

The application of LLMs in CCM is a relatively innovative field, but research is still lacking, and the overall number of papers is relatively small. Finally, 41 papers met all the inclusion criteria and were chosen for this review. Table 1 documents the research contents and publication details of the included studies. The study design and model performance details of the included studies are in Table S3 in Multimedia Appendix 1, where metrics (such as area under the receiver operating characteristic curve and area under the precision-recall curve, and *F*_1_-score) are described, together with the setting, implementation effect on patient outcomes, validation design, and external-validation environment.

**Table 1:** Research contents and publication details of the included studies in this review.

Authors	Published year	Article type	Study design	Journal or conference name	Country	Model	Research contents
Savage et al [68]	2023	Original research	Retrospective	*JMIR Medical Informatics*	United States	BioMed-RoBERTa	Developed and validated a language model-based screening tool for optimizing Best Practice Alerts.
Levin et al [17]	2024	Original research	Retrospective	*International Journal of Nursing Studies*	Israel	ChatGPT-4 and Claude-2 (Anthropic PBC)	Compared LLMs^a^ to neonatal nurses in clinical decision support for neonatal care.
Pham et al [69]	2024	Original research	Retrospective	*Journal of Medical Internet Research*	United States	ChatGPT-3.5 and ChatGPT-4	Evaluated LLMs in simulations for cardiac arrest and bradycardia based on the American Heart Association’s Advanced Cardiovascular Life Support guidelines.
Huespe et al [70]	2023	Observational study	Retrospective	*Critical care explorations*	Argentina	ChatGPT-3.5	Evaluated the capabilities of LLMs in generating the background sections of critical care clinical research questions compared to human researchers.
Si et al [71]	2019	Original research	Retrospective	*Journal of the American Medical Informatics Association*	United States	ELMo and BERT	Applied contextual embeddings to enhance clinical concept extraction from medical texts.
Almazyad et al [72]	2023	Original research	Retrospective	*Cureus*	Saudi Arabia	ChatGPT-4	Used LLMs to enhance expert panel discussions at a medical conference, focusing on pediatric palliative care and ethical decision-making scenarios.
Chung et al [73]	2024	Original research	Retrospective	*JAMA^b^ surgery*	United States	ChatGPT-4 Turbo	Evaluated the performance of LLMs in perioperative risk stratification and prognostication across various tasks.
Abdullahi et al [74]	2024	Original research	Retrospective	*JMIR Medical Education*	United States	Bard (Google LLC), ChatGPT-3.5, and ChatGPT-4	Assessed the effectiveness of LLMs in diagnosing rare and complex medical conditions, focusing on improving medical education and diagnostic accuracy.
Benboujja et al [75]	2024	Original research	Retrospective	*Frontiers in Public Health*	United States	ChatGPT-4	Developed and applied a multilingual, AI^c^-driven educational curriculum in pediatric care to overcome language barriers in global health care education.
Lu et al [1]	2023	Letter	Retrospective	*Annals of Biomedical Engineering*	China	ChatGPT-3.5 and ChatGPT-4	Explored potential uses of LLMs in intensive care medicine, focusing on knowledge augmentation, device management, clinical decision support, early warning systems, and ICU^d^ database establishment.
Madden et al [76]	2023	Letter	Retrospective	*Intensive Care Medicine*	Ireland	ChatGPT-4	Evaluated the effectiveness of LLMs in querying and summarizing unstructured medical notes in the ICU.
Liu et al [77]	2024	Original research	Prospective	*Heliyon*	China	ChatGPT-3.5 and ChatGPT-4	Assessed LLMs in predicting the risk of endotracheal intubation after initiating high-flow oxygen therapy, highlighting potential in decision-making in critical care.
Nolan et al [78]	2024	Original research	Retrospective	*Critical Care Explorations*	United States	ChatGPT-3.5 and BERT	Investigated the use of LLMs in assisting surrogate and proxy decision-making in critical care, focusing on aligning treatment recommendations with patient values.
Shah-Mohammadi and Finkelstein [79]	2024	Original research	Retrospective	*JMIR Medical Informatics*	United States	ChatGPT-3.5	Investigated ChatGPT-3.5’s application in extracting substance use information from ICU discharge summaries, highlighting improvements in accuracy with different learning scenarios.
Nawab et al [80]	2024	Original research	Retrospective	*Journal of Medical Artificial Intelligence*	United States	ChatGPT-3.5 Turbo	Evaluated ChatGPT-3.5 Turbo’s effectiveness in assigning *ICD-10*^e^ codes to clinical notes, demonstrating improved accuracy with fine-tuning, particularly in critical care administrative tasks.
Soleimani et al [81]	2024	Original research	Retrospective	*Academic Radiology*	Iran	ChatGPT-3.5 and Claude.ai	Assessed ChatGPT’s ability to generate radiology reports using MIMIC-CXR^f^ data, with a 3-step prompt guiding report synthesis. Compared outputs to Bart and XLM, showing high similarity to human reports.
Oh et al [82]	2024	Original research	Prospective	*Healthcare Informatics Research*	Korea	ChatGPT-3.5 and ChatGPT-4	Analyzed ChatGPT-3.5-turbo and ChatGPT-4 in predicting sepsis mortality using data from the Korean Sepsis Alliance.
Urquhart et al [83]	2024	Original research	Retrospective	*Intensive Care Medicine Experimental*	Ireland	ChatGPT-3.5, ChatGPT-4, and Llama-2 (Meta)	Evaluated ChatGPT-4, ChatGPT, and Llama 2 for generating ICU discharge summaries, emphasizing event recall and readability.
Pabon et al [84]	2024	Original research	Retrospective	*European Journal of Heart Failure*	United States	ChatGPT-3.5	Explored in-hospital outcomes for heart failure patients with improved ejection fraction. ChatGPT-3.5 was used to extract LVEF^g^ data from medical records, but incomplete data detection in some cases required manual review.
Akhondi-Asl et al [85]	2024	Original research	Retrospective	*Pediatric Critical Care Medicine*	United States	Llama-7B, Llama-65B, and BioGPT-Large	Evaluated domain-specific fine-tuned models against general LLMs for generating differential diagnoses in PICU^h^ patients. Fine-tuned Llama-7B outperformed larger models, demonstrating the importance of domain-specific training.
Liu et al [86]	2025	Original research	Retrospective	*Clinical Simulation in Nursing*	China	ChatGPT-3.5	Qualitative exploration of ICU novice simulation instructors’ experience with ChatGPT in case design, focusing on perceived value, potential applications, and limitations.
Berger et al [87]	2025	Original research	Retrospective	*Journal of Critical Care*	Switzerland	ChatGPT-4o	Qualitative investigation of abbreviation uses in ICU communication, focusing on risks, clinician perceptions, and patient safety implications.
Kurz et al [88]	2025	Original research	Retrospective	*NPJ Digital Medicine*	Germany	DeepSeek, InternVL, and ChatGPT-4o	Comparative benchmarking of LLMs for diagnostic accuracy using medical images plus clinical context in emergency and critical care.
Pham et al [89]	2025	Original research	Retrospective	*Cureus*	Vietnam	ChatGPT-4o	Assessment of ChatGPT-4o’s ability to interpret cranial ultrasound images for PV-IVH^i^ diagnosis in very preterm infants, compared to pediatric radiologists.
Shi et al [90]	2025	Original research	Retrospective	*Journal of Medical Internet Research*	China	SWEDEHEART-AI, Qwen-2, and Llama-3	Comparison of LLMs for predicting 1-year all-cause mortality post-AMI^j^, using structured variables versus discharge note analysis.
Yitzhaki et al [91]	2025	Original research	Retrospective	*Journal of Paediatrics and Child Health*	Israel	ChatGPT-4o	Comparative evaluation of ChatGPT-4 versus PICU specialist in answering open-ended medical education questions sourced from a trainee WhatsApp (WhatsApp LLC) forum.
Williams and Erstad [92]	2025	Original research	Retrospective	*American Journal of Health-System Pharmacy*	United States	ChatGPT-4, Copilot (Microsoft Corp), Gemini 1.5, and Meta AI	Evaluation of 4 LLMs’ responses to SCCM^k^ guideline–based medication questions.
Yang et al [93]	2025	Original research	Retrospective	*JMIR Medical Informatics*	China	ICU-GPT^l^	Development of an automated deployment and extraction platform to allow SQL generation and data retrieval from ICU-related databases without coding.
Workum et al [94]	2025	Original research	Retrospective	*Critical Care*	Netherlands	ChatGPT-4o, ChatGPT-4o-mini, ChatGPT-3.5-turbo, Mistral Large 2407, and Llama-3.1 70B	Benchmarking 5 LLMs using expert-level ICU MCQs^m^, compared against human physicians and random guessing.
Yang et al [95]	2025	Original research	Retrospective	*Frontiers in Artificial Intelligence*	United States	ChatGPT-3.5, ChatGPT-4, Claude 2, Llama2-7B, and Llama2-13B	Comparative evaluation of 5 LLMs on multiple-choice questions in critical care pharmacotherapy education, including prompt-engineering effects and a custom GPT.
Ding et al [96]	2024	Original research	Retrospective	*Scientific Reports*	United States	BlueBERT	Developed a framework that distills LLM knowledge into structured multimodal EHR^n^ predictive models for ICU health event prediction.
Walker et al [97]	2025	Original research	Retrospective	*Journal of the American Medical Informatics Association*	United States	ChatGPT-3.5	Development of CARE-SD: supervised classifiers to detect stigmatizing and doubt-marker language in ICU clinical notes using lexicon- and model-based NLP^o^.
Chen et al [98]	2025	Original research	Retrospective	*BMC Medical Education*	United States	DeepL, Gemini (Google LLC), Google Translate, and Microsoft Copilot	Multimodal assessment of freely available MT^p^ tools translating critical care educational content into Chinese, Spanish, and Ukrainian.
Ucdal et al [99]	2025	Original research	Retrospective	*Journal of Clinical Medicine*	Turkey	Gemini	Evaluation of Gemini’s application of ACG^q^ 2024 guidelines to diagnose severity and guide management in acute pancreatitis.
Balta et al [100]	2024	Original research	Retrospective	*Journal of Intensive Care Medicine*	Canada	ChatGPT-3.5 and ChatGPT-4	Comparative evaluation of ChatGPT-3.5 versus 4.0 on appropriateness, consistency, and readability of critical care recommendations.
Zhu et al [101]	2025	Original research	Retrospective	*BME Frontiers*	China	ChatGPT-4o and ChatGPT-4o mini	Evaluation of contextualized versus static word embeddings in predicting AKI^r^ using ICU clinical notes and structured data, via CNN^s^ models.
Pathak et al [102]	2025	Original research	Retrospective	*IEEE Journal of Biomedical and Health Informatics*	United States	RespBERT	Development and evaluation of RespBERT that identifies ARDS^t^ from radiology report texts using BERT embeddings and transfer learning.
Liu et al [103]	2025	Original research	Retrospective	*IEEE Journal of Biomedical and Health Informatics*	Australia	ChatGPT-4o	Development of a note-specific hierarchical network for predicting ICU in-hospital mortality from clinical notes; compares against supervised baselines and LLMs using diverse prompting strategies.
Turan et al [104]	2025	Original research	Prospective	*Journal of Clinical Anesthesia*	Turkey	ChatGPT-4	Prospective evaluation of ChatGPT-4 in interpreting ABG^u^ test results compared to expert anesthesiologists.
Wang et al [105]	2025	Original research	Retrospective	*Journal of Critical Care*	China	ChatGPT-4	Evaluation of ChatGPT-4’s performance on the Chinese critical care physician qualification examination covering multiple domains.
Yang et al [106]	2025	Original research	Retrospective	*Journal of Medical Internet Research*	China	ChatGPT-4, Qwen-2, and Llama-3	LLM-driven extraction of entities and relations to build a sepsis knowledge graph using multicenter clinical data.

^a^LLM: large language model.

^b^JAMA: *Journal of the American Medical Association*.

^c^AI: artificial intelligence.

^d^ICU: intensive care unit.

^e^*ICD-10: International Statistical Classification of Diseases, Tenth Revision*.

^f^MIMIC-CXR: Medical Information Mart in Intensive Care-Chest X-Ray.

^g^LVEF: left ventricular ejection fraction.

^h^PICU: pediatric intensive care unit.

^i^PV-IVH: periventricular-intraventricular hemorrhage.

^j^Post-AMI: postacute myocardial infarction.

^k^SCCM: Society of Critical Care Medicine.

^l^ICU-GPT: Intensive Care Unit-specific Generative Pre-trained Transformer.

^m^MCQ: multiple choice question.

^n^EHR: electronic health record.

^o^NLP: natural language processing.

^p^MT: machine translation.

^q^ACG: American College of Gastroenterology.

^r^AKI: acute kidney injury.

^s^CNN: convolutional neural network.

^t^ARDS: acute respiratory distress syndrome.

^u^ABG: arterial blood gas.

### Bibliometric Analysis

This scoping review included a focused selection of 41 papers, providing a global perspective on LLM applications in CCM. This diverse corpus spans several countries, demonstrating widespread research interest in applying LLMs in CCM. The results of keyword co-occurrence network analysis are in Note S4 in Multimedia Appendix 1. Among the 41 papers, only 2 used prospective data, while the other 39 were retrospective studies. The distribution of the selected publications indicates substantial international collaboration and research efforts. We analyzed the countries where each selected paper’s first and corresponding authors were based. The authors from the United States took the lead in most studies, followed by authors from China, Ireland, Israel, Korea, etc. It revealed that nearly half of the studies were conducted in the United States, with much fewer contributions from other countries and regions. This indicates a concentration of research activities in applying LLMs in CCM within the United States, potentially reflecting the advanced development and adoption of AI technologies in American critical care settings. Among the LLMs used, ChatGPT-4 appears most frequently, demonstrating its relevance and recent prominence in CCM applications. Other models include ChatGPT-3.5 and models such as Llama, Gemini, Claude, and DeepSeek, highlighting the breadth of generative models explored in the included studies. A minority of studies use domain-adapted or clinical NLP backbones, including BioGPT-Large, BioMed-RoBERTa, BlueBERT, and RespBERT.

### Applications of LLMs in CCM

#### Clinical Decision Support

**Figure 2:**
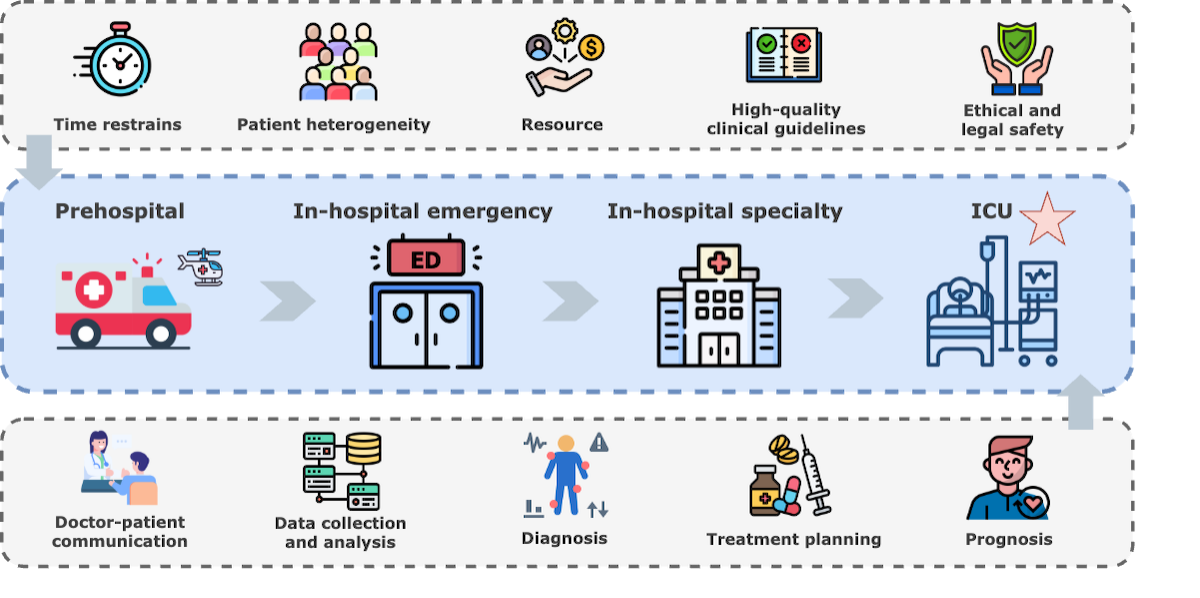
The process and characteristics of clinical decision support. ED: emergency department; ICU: intensive care unit.

As illustrated in Figure 2, the primary application of LLMs in CCM is clinical decision support. LLMs can be applied in diagnosis, treatment planning, and prognosis prediction in in-hospital critical care settings.

In diagnosis, LLMs demonstrate the potential to aid physicians in making diagnostic decisions. Gandomi et al [107] explored the use of LLMs (Llama 70B and Mistral) in detecting ARDS from radiology reports in the MIMIC-III (Medical Information Mart for Intensive Care-III) database. The study applied LLMs to identify high-probability ARDS cases based on bilateral infiltrates. Akhondi-Asl et al [85] investigated the use of domain-specific fine-tuned models (Llama-7B and BioGPT-Large) against larger general-domain models (Llama-65B) for generating differential diagnoses in pediatric intensive care unit (PICU) patients. They found that fine-tuned Llama-7B outperformed both Llama-65B and BioGPT-Large. Kurz et al [88] benchmarked the diagnostic performance of various vision-language models on a multimodal dataset comprising medical images and associated clinical context in ICU environments. They compared several open-source vision-language models against ChatGPT-4o, finding that while open models achieved accuracy only up to 40.4%, ChatGPT-4o substantially outperformed them with an approximation of 68.1%. Pham et al [89] evaluated the diagnostic utility of ChatGPT-4o in interpreting cranial ultrasound images to detect periventricular-intraventricular hemorrhage among very preterm infants in a neonatal ICU in Vietnam. Comparing ChatGPT-4o’s image-based diagnoses against pediatric radiologists, the model achieved moderate performance (area under the curve [AUC]=0.796), with 75% sensitivity and 84.2% specificity, alongside fair-to-good interrater agreement. The study highlights ChatGPT-4o’s potential as a supplemental tool for early periventricular-intraventricular hemorrhage screening. Pathak et al [102] developed RespBERT, leveraging BERT-based embedding and transfer learning to automatically identify ARDS from unstructured radiology notes across multiple hospital datasets. Applying the model to notes from 2 independent institutions, RespBERT achieved *F*_1_-scores of 74.5% and 64.2%, demonstrating robust performance across different clinical settings and indicating its potential for ARDS detection in the ICU.

In treatment planning, LLMs show considerable promise in providing personalized treatment recommendations and optimizing clinical pathways for patients with critical illness. Savage et al [68] developed and validated the LLM screening tool to selectively identify patients appropriate for Best Practice Alerts of deep vein thrombosis anticoagulation prophylaxis using the MIMIC-III database. They found that the LLM screening tool improved the precision of Best Practice Alerts, reducing the number of unnecessary alerts by 20% and increasing the applicability of alerts by 14.8%. Pham et al [69] evaluated ChatGPT’s performance in treating cardiac arrest and bradycardia simulations in accordance with the American Heart Association’s Advanced Cardiovascular Life Support guidelines. Using the 2020 Advanced Cardiovascular Life Support guidelines, ChatGPT’s responses to 2 simulation scenarios were assessed for accuracy. They found that ChatGPT had a median accuracy of 69% for cardiac arrest and 42% for bradycardia, with significant variability in its outputs, often missing critical actions, and having incorrect medication information. Nolan et al [78] used LLMs to support critical care decision-making for incapacitated patients. The study simulated scenarios for 50 patients requiring urgent clinical decisions and incorporated patient values captured through various formats, including free-text narratives. The LLMs were tasked with extracting treatments and generating recommendations based on patient profiles. The results showed that LLMs accurately extracted treatments in 88% of cases and received high scores for providing medically plausible and value-aligned recommendations. Williams et al [92] evaluated 4 LLMs (ChatGPT-4, Copilot, Gemini version 1.5, and Meta AI) by medication-related questions based on 6 Society of Critical Care Medicine clinical practice guidelines. Copilot yielded the highest proportion of correct answers, followed by Meta AI, ChatGPT-4, and Gemini, which delivered the most incorrect responses. Despite these capabilities, none of the models consistently matched guideline recommendations, indicating that while clinically promising, AI tools require further development for reliable use in the ICU. Ucdal et al [99] assessed the performance of Gemini in applying American College of Gastroenterology 2024 guidelines to clinical decisions in acute pancreatitis management. Using MIMIC-III data consisting of 512 patient cases, the study evaluated Gemini’s accuracy in determining disease severity, recommending nutritional strategies, and antibiotic use. Gemini achieved 85% accuracy for mild cases, 82% for severe cases, and 78%-85% compliance with guideline-based nutritional and management recommendations, demonstrating solid agreement with scoring systems. This suggests that AI may support consistent, guideline-concordant decision-making in pancreatitis care. Turan et al [104] conducted a prospective observational study comparing ChatGPT-4’s interpretation of arterial blood gas results with that of expert anesthesiologists using 400 ICU patient samples. The model demonstrated excellent accuracy for parameters such as pH, oxygenation, sodium, chloride, and hemoglobin, though it struggled with bilirubin. While generally reliable, it occasionally recommended unnecessary bicarbonate therapy, highlighting the promise of ChatGPT-4 as a rapid interpretive aid, but underscoring the necessity of clinician oversight. Yang et al [106] presented a pioneering study that leveraged ChatGPT-4 and a multicenter real-world sepsis dataset (10,544 patients across 3 hospitals in China) to construct a comprehensive sepsis knowledge graph. By combining clinical guidelines, public data, and advanced prompt engineering, they extracted entities and relationships, building a graph with 1894 nodes and 2021 unique connections. ChatGPT-4 achieved a high *F*_1_-score of 76.76 on the study’s sepsis-specific dataset and 65.42 under the few-shot condition, surpassing models such as Qwen-2 and Llama-3.

For prognosis prediction, Amacher et al [108] used ChatGPT-4 to predict mortality and poor neurological outcomes at hospital discharge for adult patients who had cardiac arrest. The study involved prompting ChatGPT-4 with 16 prognostic parameters from established post–cardiac arrest scores. The findings showed that ChatGPT-4 achieved an AUC of 0.85 for in-hospital mortality and 0.84 for poor neurological outcomes, comparable to traditional scoring systems. Chung et al [73] used ChatGPT-4 to perform risk stratification and predict postoperative outcomes based on procedure descriptions and preoperative clinical notes from electronic health records (EHRs). They found that ChatGPT-4 achieved *F*_1_-scores of 0.64 for predicting hospital admission, 0.81 for ICU admission, 0.61 for unplanned admission, and 0.86 for predicting hospital mortality. Liu et al [77] conducted a prospective multicenter cohort study using ChatGPT-3.5 and ChatGPT-4 to predict the risk of endotracheal intubation within 48 hours following high-flow nasal cannula oxygen therapy in patients with critical illness. They found that ChatGPT-4 achieved an accuracy comparable to that of specialist physicians, with an AUC of 0.82, which was higher than that of non-specialist physicians (AUC=0.66). Oh et al [82] conducted a study using ChatGPT-3.5-turbo and ChatGPT-4 to predict in-hospital mortality for sepsis patients. The study used clinical data from the Korean Sepsis Alliance database, focusing on ICU admissions and using metrics such as the SOFA (Sequential Organ Failure Assessment) score and lactic acid levels. The findings demonstrated that ChatGPT-4 performed comparably to a gradient boosting machine in predicting short-term mortality, particularly for 7-day outcomes. Shi et al [90] used the MIMIC-IV (Medical Information Mart for Intensive Care-IV) database to compare the performance of 2 LLMs (Qwen-2 and Llama-3) with a specialized artificial neural network (SWEDEHEART-AI) trained on Swedish registry data, in predicting 1-year all-cause mortality among ICU patients with acute myocardial infarction. SWEDEHEART-AI outperformed both LLMs, maintained consistent area under the receiver operating characteristic curve in time-dependent analyses, and demonstrated superior clinical utility and net benefit across risk thresholds, suggesting its stronger reliability for risk stratification. Ding et al [96] proposed a novel framework, cross-modality knowledge learning and extraction, that distills knowledge from LLMs into a predictive model trained on multimodal EHR data in the ICU. By refining clinical text using LLM-generated embeddings and using a cross-modality knowledge distillation approach that combines contrastive and patient-similarity learning losses, cross-modality knowledge learning and extraction significantly improved predictive accuracy for hypertension and heart failure events, demonstrating up to a 4.48% boost over state-of-the-art models using data from the MIMIC-III database. Zhu et al [101] used clinical data from 2 Chinese hospitals and a public South Korean dataset, comprising a total of 2649 older adult patients who underwent surgery, to use LLMs (GPT-4o, ChatGPT-4o mini, Qwen2-7B-Instruct, and Llama3.1-8B-Instruct), comparing their performance against traditional ML models such as XGBoost (Extreme Gradient Boosting) and Random Forest for the task of predicting postoperative AKI. The study enhanced the LLMs’ capabilities through prompt engineering techniques such as Medical Chain of Thought and instruction fine-tuning. The results demonstrated that the LLM-based frameworks achieved superior generalization on external datasets while also providing human-readable medical rationales for predictions, significantly improving interpretability and clinical utility compared to traditional ML approaches. Liu et al [103] investigated risk prediction of in-hospital mortality using routinely collected clinical notes in the ICU. It proposes a note-specific hierarchical network that adapts to different note types and benchmarks it against various supervised baselines and 34 instruction-following LLMs under zero-shot and few-shot settings, as well as chain-of-thought prompting. The hierarchical model outperformed both LLMs and supervised baselines, which consistently underperformed in this critical task, highlighting important constraints of LLMs in risk assessment of critical care patients.

#### Medical Documentation and Reporting

LLMs are making strides in medical documentation and reporting by automating and streamlining these processes. Shah-Mohammadi et al [79] used the ChatGPT-3.5 model to extract substance use information from ICU discharge summaries in the MIMIC-III database, focusing on tobacco, alcohol, and illicit substances. They explored both zero-shot and few-shot prompt learning settings and found that GPT’s performance in identifying tobacco, alcohol, and substance use varied depending on the learning scenario. Zero-shot learning achieved high accuracy in recognizing substance use, while few-shot learning, although lowering accuracy, improved the identification of substance use status, leading to better recall and *F*_1_-scores but lower precision. Nawab et al [80] conducted a study using the ChatGPT-3.5 Turbo to automate the assignment of *ICD-10* codes to clinical notes in the ICU. Their findings demonstrated that fine-tuning the model with a specialized dataset improved its accuracy from 29.7% to 62.6%. Soleimani et al [81] conducted a study using ChatGPT-3.5 to evaluate the performance of radiology report generation. Using data from the MIMIC-CXR (Medical Information Mart for Intensive Care-Chest X-Ray) database, the study explored how ChatGPT, guided by a 3-step prompt, synthesized complete radiology reports. They found that ChatGPT effectively generated comprehensive reports by accurately interpreting both patient characteristics and radiological findings. Urquhart et al [83] used ChatGPT-4, ChatGPT-3.5, and Llama 2 to extract key information from ICU patient text records in an Irish population. The study evaluated the models’ ability to generate concise and accurate clinical summaries from unstructured ICU admission notes. The results showed that ChatGPT-4 outperformed the other models in readability, organization, and summarization of clinically significant events, but all models struggled with completeness and narrative coherence. Pabon et al [84] used ChatGPT-3.5 for extracting left ventricular ejection fraction data from medical records in a study involving patients with heart failure with improved ejection fraction. The model achieved 100% accuracy in identifying reported left ventricular ejection fraction values but struggled with a capture completeness of 75%. Si et al [71] explored the impact of ELMo and BERT on clinical concept extraction tasks using data from the MIMIC-III and other clinical corpora. They found that contextual embeddings pretrained on a large clinical corpus outperformed traditional methods. Madden et al [76] used ChatGPT-4 to query and summarize unstructured medical notes in the ICU. They found that while the model could produce concise and useful summaries, it also had significant risks of generating hallucinations. Yang et al [93] developed a platform to facilitate the deployment and extraction of critical care-related big data using LLMs. The system leverages Docker (Docker Inc)–based automated database deployment and visualization tools, along with an ICU-fine-tuned LLM ICU-GPT to generate SQL queries and extract data from complex ICU datasets without requiring programming knowledge. This platform enables clinicians to manage, visualize, and retrieve structured insights from large critical care databases through a user-friendly web interface, reducing the technical barrier to big data research in clinical settings. Walker et al [97] developed CARE-SD, a classifier-based NLP toolkit designed to identify stigmatizing patient labels and doubt markers within ICU clinical notes. By constructing lexicons (127 stigmatizing expressions and 58 doubt markers) using literature-based stems augmented via Word2Vec and ChatGPT-3.5, and training supervised classifiers on annotated samples drawn from 18 million MIMIC-III sentences, the models achieved macro *F*_1_-scores of 0.84 (doubt markers) and 0.79 (stigmatizing labels). This approach supports the detection of linguistic biases in critical care EHRs and could inform interventions to reduce stigmatizing language in health care.

#### Medical Education and Doctor-Patient Communication

LLMs are used more and more frequently in medical education now. One important area closely connected to LLMs is to generate or answer questions in medical examinations. Workum et al [94] conducted a benchmark study by evaluating 5 LLMs (GPT-4o, ChatGPT-4o-mini, ChatGPT-3.5-turbo, Mistral Large 2407, and Llama 3.1 70B) on 1181 multiple-choice questions from the European Diploma in Intensive Care examination. All models significantly outperformed human physicians, with ChatGPT-4o achieving the highest accuracy of 93.3%. Despite outstanding consistency and performance, models still produced incorrect answers and raised concerns about energy consumption, especially for ChatGPT-4o, highlighting the need for ongoing evaluation before clinical deployment. Yang et al [95] compared the performance and consistency of 5 LLMs (GPT-3.5, ChatGPT-4, Claude 2, Llama2-7B, and Llama2-13B) on a set of 219 multiple-choice questions covering critical care pharmacotherapy for Doctor of Pharmacy students. The study evaluated accuracy, response variance, and the impact of prompt engineering techniques, such as few-shot chain-of-thought prompting, and the use of a custom-trained GPT model. ChatGPT-4 emerged with the highest accuracy (71.6%), chain-of-thought prompting further improved its performance, and the variance in performance differed across models. Notably, customizing models and prompt strategies can enhance LLM reliability in pharmacy education contexts. Chen et al [98] developed and applied a multimodal evaluation framework to assess the performance of widely available machine translation (MT) tools (including DeepL, Gemini, Google Translate, and Microsoft Copilot) in translating critical care educational content from English into Mandarin Chinese, Spanish, and Ukrainian. The study used blinded bilingual clinician ratings (for fluency, adequacy, and meaning), BLEU (bilingual evaluation understudy) scores, and usability assessments to compare MT outputs against professional human translations. The results revealed no single MT tool consistently excelled across languages or metrics, human translation scored best for Chinese, Gemini performed strongest for Spanish, and Microsoft Copilot ranked highest for Ukrainian, highlighting the need for ongoing evaluation of MT tools in critical care education as they rapidly evolve. Wang et al [105] evaluated ChatGPT-4 against the Chinese Health Professional Technical Qualification Examination for Critical Care Medicine, which comprises 600 questions across fundamental knowledge, specialized knowledge, practical skills, and related medical knowledge. ChatGPT-4 achieved an overall success rate of 73.5%, surpassing the 60% passing threshold, with the highest accuracy in fundamental knowledge (81.94%). Notably, performance was significantly better on single-choice versus multiple-choice questions (76.72% vs 51.32%, *P*<.001), with no difference between case-based and non–case-based formats. The study underscores its potential as a clinical decision support and educational aid, while cautioning on the need for expert oversight due to potential inaccuracies.

Meanwhile, LLMs use information such as clinical guidelines to answer questions and do clinical reasoning from real-world medical scenarios. Levin et al [17] used 2 LLMs, ChatGPT-4 and Claude-2.0, to provide initial assessment and treatment recommendations for patients in neonatal intensive care settings. The results indicated that both models demonstrated clinical reasoning abilities, with Claude-2.0 outperforming ChatGPT-4 in clinical accuracy in providing initial assessments and treatment recommendations, and response speed. Liu et al [86] conducted a qualitative study to explore how novice ICU simulation instructors experience the use of GenAI in case design. Using semistructured interviews with 13 instructors and thematic analysis, the study found that GenAI improved efficiency, provided structured and diverse scenario design, and enhanced learning engagement, especially for beginners. The findings suggest that GenAI can serve as a valuable educational tool in ICU simulation, but must be balanced with instructor-led critical thinking and validated clinical accuracy. Yitzhaki et al [91] used 100 educational questions from a PICU trainee WhatsApp forum to compare ChatGPT-4’s performance against pediatric intensive care specialists. Evaluated by 10 PICU experts across multiple tertiary centers, ChatGPT-4’s responses were longer and more complete for factual questions, with 60% being preferred for factual knowledge; however, specialists’ responses were favored in clinical reasoning (67%), reflecting higher accuracy. Integrated answers were chosen in 37% of evaluations, emphasizing the need for expert oversight when using ChatGPT-4 in PICU education. Balta et al [100] assessed ChatGPT-3.5 versus ChatGPT-4 by having 2 independent intensivists evaluate LLM-generated recommendations to 50 curated core critical care questions from textbooks. ChatGPT-4 delivered significantly higher median appropriateness scores. The study stresses that both models can confidently produce clinically misleading or hallucinated content and thus should be used with caution in the ICU.

LLMs can also be used to overcome language barriers and enhance communication. Benboujja et al [75] developed and evaluated a multilingual, AI-driven curriculum to overcome language barriers in pediatric care. Using ChatGPT-4 for translation, the study created 45 educational video modules in English and Spanish, covering surgical procedures, perioperative care, and patient journeys. Almazyad et al [72] used ChatGPT-4 to enhance expert panel discussions in pediatric palliative care. They found that ChatGPT-4 effectively facilitated discussions on do-not-resuscitate conflicts by summarizing key themes such as communication, collaboration, patient and family-centered care, trust, and ethical considerations. Berger et al [87] conducted a study to investigate the risks associated with the use of abbreviations in critical care communication. By analyzing perspectives from ICU clinicians, the study highlighted how abbreviations, although designed to save time, often introduce ambiguity, misinterpretation, and patient safety risks in high-stakes environments. The findings emphasize that abbreviations can fall short of their intended efficiency, underscoring the importance of clear communication, standardized language, and improved training to minimize preventable errors and improve patient safety in the ICU.

#### RoB and Applicability Assessment

Across the literature, most studies were judged to have a high RoB in at least 1 RoB domain, most commonly analysis, including reliance on apparent performance without robust internal validation, absent calibration or uncertainty, and risks of temporal or selection leakage, followed by predictors and participants. Outcome definitions were generally aligned with ICU standards, but sometimes lacked blinded ascertainment. Applicability concerns concentrated in predictors (including dependencies on sources not readily available in real-time ICU workflows). Detailed ratings and justifications are presented in Table S4 in Multimedia Appendix 1.

## Discussion

### Principal Findings

**Figure 3:**
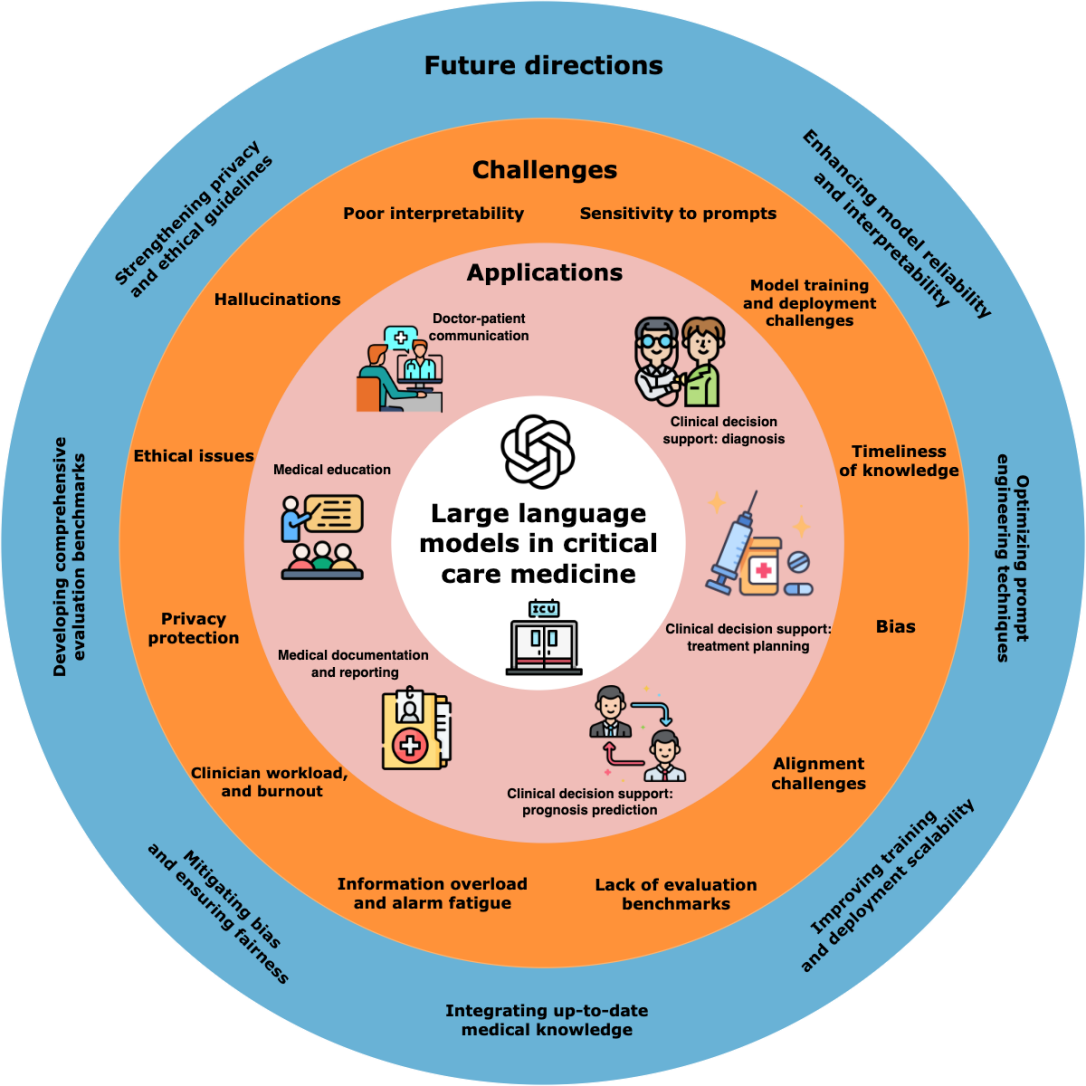
The main applications, challenges, and future directions of LLMs in CCM. CCM: critical care medicine; LLM: large language model.

This scoping review provided a comprehensive portrait of the role of LLMs in CCM, identifying the applications, advantages, challenges, and future research directions of this area. With the recent advent of LLMs, medicine has witnessed groundbreaking developments and advancements [109]. Many review papers focus on applying LLMs in health and medicine [110,111]. Particularly, although there are some review papers on AI in CCM [112-114], few review papers focus on the application of LLMs in CCM. From the 2342 papers initially retrieved, 41 of them were selected for final review. An extensive examination of the selected literature revealed that LLMs have shown promise in some main aspects of CCM: clinical decision support, medical documentation and reporting, medical education, and doctor-patient communication. Compared with traditional AI models, LLMs have advantages in processing unstructured data and do not require manual feature engineering. At the same time, applying LLMs to CCM faces numerous challenges, including hallucinations and poor interpretability, sensitivity to prompts, bias and alignment challenges, and privacy and ethical issues. The current applications, together with the challenges and future directions of LLMs in CCM identified by this review, are shown in Figure 3. Our findings highlight the potential of LLMs in critical care practices while also underscoring the need for further research to address corresponding challenges and improve the reliability and applicability of LLMs in the critical care domain.

The application of LLMs in CCM has demonstrated numerous advantages. Compared to traditional machine learning techniques, using LLM technology in CCM can effectively cause understanding and generate natural language, aiding clinicians in writing patient medical records and diagnostic notes [115-118]. The capabilities of LLMs extend beyond text interpretation and generation. They surpass traditional machine learning methods in handling unstructured data. LLMs can learn directly from extensive patient data without manual feature engineering. Moreover, multimodal LLMs can learn and understand medical images, such as x-rays and CT (computed tomography) scans [110]. In clinical practice, LLMs can extract critical information from a patient’s historical medical records and combine it with the latest medical research, aiding physicians in identifying rare diseases or those with early symptoms that are not clearly defined [119]. For medical research, LLMs can assist researchers in summarizing data and information in literature research and providing suggestions for manuscript structure and titles, enhancing the readability and completeness of texts [118]. LLMs have a wide range of knowledge that can provide physicians with a comprehensive analysis of decision-making across different specialties [115].

### Challenges

#### Hallucinations and Poor Interpretability

One of the most critical challenges in applying LLMs in CCM is the occurrence of hallucinations, where the model generates plausible-sounding but factually incorrect information [120,121]. These hallucinations pose risks, where incorrect recommendations can lead to inappropriate diagnoses or treatment plans, potentially endangering ICU patients’ lives. Studies have documented examples where LLMs hallucinate during critical care decision-making, raising concerns about their reliability [76,81,122]. Additionally, LLM outputs often lack transparency, making it difficult for clinicians to understand how specific decisions are reached. This opacity complicates the tracking of decision-making processes, leading to reduced trust in these systems [61,123]. The opaque nature of LLMs further complicates their application, as it becomes challenging to verify the factual basis of their outputs, particularly in the ICU, where patient safety is important. The lack of clarity regarding the sources of information makes LLMs unreliable for autonomous decision-making in CCM [76,81]. Therefore, improving the interpretability and transparency of LLMs remains a critical challenge in their integration into clinical workflows [123,124].

#### Sensitivity to Prompts and Inconsistent Performance

LLM-generated outputs are highly sensitive to input prompts, and different prompt strategies may affect the model’s capabilities and performance [122]. In CCM, where the accuracy of information is essential, this sensitivity can lead to inconsistent results across different clinical scenarios [82,84]. The same LLM might provide different answers to slightly rephrased prompts, requiring clinicians to analyze multiple iterations to ensure accuracy. This lack of consistency raises concerns about the reliability of LLM applications in critical care, especially when time-sensitive decisions must be made. There is no universal prompt strategy that guarantees high performance across all LLMs, which means that clinicians must carefully craft prompts to suit the context of the task [75]. The absence of a one-size-fits-all approach for prompting remains a significant limitation of LLMs in critical care settings [76].

#### Model Training and Deployment Challenges

Training and deploying LLMs for critical care applications is a resource-intensive process, requiring vast computational power and large, diverse datasets. Public critical care databases, such as MIMIC (Medical Information Mart for Intensive Care) [125,126] and eICU [127], are commonly used for model training. Moreover, hospital regulations and privacy laws often restrict data sharing, complicating the training of models across multiple centers [107]. The deployment of LLMs in real-time ICU settings also requires substantial computational resources, which may not be feasible in all clinical environments, particularly those with constrained infrastructure [68,128,129]. Additionally, compliance with local data privacy regulations in some hospitals can limit the use of LLMs in health care settings [107]. The inability of LLMs to adapt to local computing environments due to privacy concerns further hampers their wide-scale application in critical care [68].

#### Timeliness of Knowledge and Model Updates

CCM is a rapidly evolving field, with frequent updates to clinical guidelines, treatments, and best practices. LLMs, however, are typically trained on static datasets, limiting their ability to remain up-to-date with the latest medical advancements [19]. This delay in knowledge poses a significant challenge, as outdated information could negatively impact patient outcomes [76,84]. To ensure their continued relevance in clinical practice, LLMs must be regularly updated with the latest medical knowledge. However, this process is computationally expensive and logistically challenging, particularly for large-scale LLMs [84,130]. Therefore, ensuring the timeliness of LLMs’ knowledge base is crucial to their successful application in CCM.

#### Bias and Alignment Challenges

LLMs may unintentionally learn biases from the training data and reproduce them in their outputs, potentially leading to skewed or inappropriate recommendations in critical care. These biases can disproportionately affect specific patient populations, potentially leading to disparities in treatment and care [70,78,131]. In the ICU, biased outputs could result in suboptimal or even harmful decisions. Moreover, aligning LLM behavior with clinical guidelines and ethical standards is also a challenge, as models may not always adhere to best practices when generating recommendations [81,84,132]. Addressing bias and ensuring alignment with clinical guidelines and ethics are essential steps for LLMs to function as reliable tools in critical care [131,133]. Additionally, excessive reliance on AI-generated alerts can lead to “alarm fatigue,” where clinicians become desensitized to frequent, nonurgent predictions, potentially missing critical care events [134].

#### Lack of Evaluation Benchmarks

Currently, there are no universally accepted standards for evaluating the performance of LLMs in critical care settings. Traditional model evaluation primarily focuses on the accuracy of medical question answering, which may not fully reflect the capabilities of LLMs in critical care clinical practice [135]. Across the included literature, model discrimination should not be conflated with clinical benefit. Consistent with current professional guidance and editorials, routine adoption should follow prospective evaluations that consider patient-centered outcomes rather than relying solely on technical metrics. Current benchmarks rarely include patient-relevant end points [136,137]. Without appropriate benchmarks, it is challenging to evaluate the effectiveness of LLMs in critical care or compare different models on an equal standard.

#### Information Overload and Alarm Fatigue in ICU Workflows

Contemporary ICUs are characterized by a proliferation of bedside devices and dense, multistream monitoring data. Qualitative and narrative evidence suggest that this technological abundance, when poorly integrated, can amplify cognitive load, desensitize clinicians to frequent alarms, and undermine situation awareness and team communication [138]. LLM-enabled systems may help by acting as context-aware filters and data-to-text summarizers that deduplicate near-identical events across monitors, ventilators, or pumps; prioritize alerts using clinical context; and attach traceable rationales for rapid verification [139,140]. However, naïve deployments could also increase burden (eg, secondary notifications and unverifiable rationales), so any use of LLMs should be aligned with human-centered monitoring principles and embedded in sociotechnical workflows rather than added as another layer.

#### Clinician Workload, Burnout, and Documentation Quality

While LLMs show promise for text generation and knowledge integration, clinical burden and professional burnout are often overlooked dimensions of the ICU setting [141,142]. Hallucinations and lack of interpretability shift the burden from “writing” to “reviewing and evidence verification.” Inconsistent sensitivity to prompts and output leads to trial and error for frontline staff [143]. At the deployment level, the ICU’s high noise level, multirole collaboration, and rigorous legal review may create new bottlenecks for editing and rework. Furthermore, language biases in generated text and paperwork bloat may compromise team communication and information retrieval efficiency. These factors collectively point to the need for systematic evaluation of clinician burden and burnout beyond model effectiveness.

#### Privacy and Ethical Concerns

Handling patient data responsibly is a significant concern in critical care, where vast amounts of sensitive information must be processed. Ensuring patient privacy while using LLMs presents both technical and legal challenges. Strict compliance with data protection regulations, such as the General Data Protection Regulation and HIPAA (Health Insurance Portability and Accountability Act), is necessary but can hinder the deployment of LLMs in clinical settings [68,76]. Moreover, the ethical implications of relying on LLMs for life-or-death decisions raise concerns about accountability and the potential over-reliance on AI in medical decision-making [144]. To navigate these issues, health care systems must establish clear guidelines for the responsible use of LLMs, ensuring that patient privacy is upheld and ethical standards are maintained. Addressing these privacy and ethical challenges will be essential for gaining clinician and patient trust in AI systems used in CCM [72].

### Future Directions

#### Enhancing Model Reliability and Interpretability

Improving the reliability and interpretability of LLMs in CCM is critical for their safe integration into real-world clinical workflows. To enhance model reliability, future research should prioritize improving the quality of training data, particularly by incorporating domain-specific knowledge from critical care environments [84,107]. The accuracy and reliability of LLMs can be enhanced by improving training data quality, using ensemble learning, evidential reasoning, implementing adversarial training, and multiagent systems [145-149]. Additionally, the use of methods, such as chain-of-thought reasoning, tree-of-thoughts, and retrieval-augmented generation (RAG), can offer greater interpretability, allowing clinicians to understand how LLMs arrive at specific recommendations [150]. These interpretability techniques would provide clinicians with a clearer rationale for decision-making, thereby building trust in LLM outputs [151,152]. LLM outputs should include provenance-linked evidence and enforce concise, structured formats to curb alert or note bloat and reduce verification burden in human-centered ICU workflows [138]. Further, integrating external knowledge databases such as PubMed through plugins can improve the accuracy of LLM outputs and reduce the risk of hallucinations, particularly in critical care [108].

#### Optimizing Prompt Engineering Techniques

LLMs are highly sensitive to prompts, and developing robust prompt engineering techniques is essential for improving consistency and reliability in CCM. Recently, advancements such as Medprompt, which combines dynamic few-shot, self-generated chain-of-thought, and choice shuffle ensemble, have demonstrated improved performance in general LLMs, particularly in medical contexts [153]. MedGraphRAG is also a novel graph-based RAG framework designed specifically for the medical domain, enhancing the capabilities of LLMs by generating evidence-based, contextually accurate responses through a hierarchical graph structure, thereby improving transparency and reliability in handling private medical data [154]. These advancements will be particularly useful for critical care environments, where fast and reliable decision-making is essential. Future research should explore the development of prompt engineering strategies to handle complex clinical tasks [74,79].

#### Improving Model Training and Deployment Scalability

To address LLM training and deployment challenges, scalable model architectures, transfer learning, model pruning, and federated learning approaches can be explored to reduce computational demands and facilitate practical deployment [155]. The emergence of low-powered open-source LLMs running locally could circumvent issues related to data privacy and computational resource constraints [76]. It is crucial to convert medical datasets into easily accessible structured databases and train health care professionals in the ICUs to use LLMs in clinical practice to aid decision-making [108]. Collaboration with hospitals to develop structured medical databases will also aid in better training of LLMs for real-time decision-making in critical care environments [84,107].

#### Integrating Up-to-Date Medical Knowledge

Using web-based learning systems allows models to update and assimilate the latest medical research and changes in clinical practices on time. Additionally, modular update systems can swiftly integrate new medical discoveries, while expert collaboration ensures the scientific validity and timeliness of model outputs. Moreover, using RAG techniques to connect LLMs with databases in CCM can also address the knowledge timeliness issue to some extent [151,152].

#### Mitigating Bias and Ensuring Fairness

Bias mitigation should be approached through preprocessing, in-training, intraprocessing, and postprocessing stages [131]. Preprocessing techniques involve modifying model inputs to ensure balanced representations. In-training methods focus on adjusting model parameters to mitigate biases through gradient-based updates. Intraprocessing methods modify inference behavior without further training, while postprocessing techniques correct model outputs to ensure fair treatment across demographic groups. Developing bias detection and dataset augmentation algorithms to review and adjust model outputs regularly can help reduce model bias and ensure fairness in CCM [156].

#### Developing Comprehensive Evaluation Benchmarks

Recent studies demonstrated that performance varies across different medical tasks, highlighting the need for task-specific evaluations [135,157]. Future efforts should focus on developing more sophisticated evaluation frameworks that go beyond traditional metrics and consider the specific challenges of critical care [68]. For instance, the MIMIC-IV-CDM (Medical Information Mart for Intensive Care-IV-Clinical Decision Making) dataset and evaluation framework, focusing on 2400 real patient cases with acute abdominal pain, offers a new benchmark for evaluating LLMs in clinical decision-making, highlighting the need for more rigorous testing to ensure LLMs meet clinical standards, particularly guidelines [158]. We must standardize clinician-centered metrics, such as time-in-note, click or keystroke counts, handoff completion time, and note quality (accuracy, completeness, consistency, and readability), and report them alongside patient outcomes in prospective, preregistered ICU studies [159]. To establish clinical benefit and safety beyond model accuracy, future studies should use preregistered, prospective designs that prespecify outcomes such as mortality, ventilator-free days, time-to-critical interventions, and ICU length of stay, alongside calibration and uncertainty reporting [136,137]. Future work should explore comparing general LLMs against domain-adapted or fine-tuned LLMs (eg, BioGPT-Large and Llama-Med) on tasks and datasets in CCM. Additionally, collaboration between medical professionals and AI researchers will be necessary to design evaluation metrics that are meaningful, clinically applicable, and capable of guiding LLM improvements [135].

#### Strengthening Privacy and Ethical Guidelines

Data privacy and ethical guidelines are crucial for ensuring that LLMs are safely integrated into CCM [123,124]. As LLMs handle vast amounts of sensitive patient data, their deployment must comply with strict data protection regulations [76]. Future research should explore synthetic data generation techniques to augment training datasets while protecting patient privacy, allowing for comprehensive model training without compromising confidentiality [76,107]. Moreover, collaboration with policymakers, ethicists, and legal experts is necessary to ensure LLM applications comply with ethical and legal requirements, thus protecting patient privacy and data security [160].

This scoping review may be limited by selection bias due to the literature databases and inclusion criteria, potentially excluding relevant studies in non-English or outside the selected databases. Additionally, the rapid development of LLMs could render the findings quickly outdated, and the broad scope may have limited the depth of analysis for specific LLM applications in CCM.

### Conclusions

In conclusion, although LLMs in CCM are not yet ICU experts, they act as more than stochastic parrots. Applying LLMs in CCM presents a transformative potential for enhancing critical care. LLMs are capable of reasoning beyond random generation, and they have demonstrated capabilities to improve diagnostic accuracy, plan optimal treatments, and provide valuable support in prognosis prediction. However, applying LLMs in CCM is still in its early stages, with very few large models specifically designed and fine-tuned for this domain. Future research should focus on enhancing model reliability and interpretability, optimizing prompt engineering techniques, improving training and deployment scalability, integrating up-to-date medical knowledge, mitigating bias and ensuring fairness, developing comprehensive evaluation benchmarks, and strengthening privacy and ethical guidelines. Close collaboration across multiple disciplines, such as medicine, computer science, and data science, may help catalyze the applications of LLMs in CCM. There is some way to go before making LLMs that become true ICU experts. Nevertheless, we are optimistic that LLMs in CCM will become experts in the near future, helping to improve the quality of critical care and the outcomes of patients with critical illness.

## Appendix

Multimedia Appendix 1 Additional materials including Note S1 (Overview of LLMs in health and medicine), Note S2 (The process for selecting sources of evidence), Note S3 (Operational rules for PROBAST-AI ratings), Note S4 (Keyword co-occurrence network), Table S1 (PRISMA-ScR checklist), Table S2 (Search terms used across multiple databases for the scoping review), Table S3 (Design and performance summary for included studies), and Table S4 (Risk of bias and applicability assessed using the PROBAST-AI). LLM: large language model; PROBAST-AI: Prediction Model Risk of Bias Assessment Tool-Artificial Intelligence.

## Abbreviations

AI: artificial intelligence
AKI: acute kidney injury
ARDS: acute respiratory distress syndrome
AUC: area under the curve
BLEU: bilingual evaluation understudy
CCM: critical care medicine
CT: computed tomography
EHR: electronic health record
GenAI: generative artificial intelligence
HIPAA: Health Insurance Portability and Accountability Act
ICU: intensive care unit
LLM: large language model
MIMIC: Medical Information Mart for Intensive Care
MIMIC-CXR: Medical Information Mart for Intensive Care-Chest X-Ray
MIMIC-III: Medical Information Mart for Intensive Care-III
MIMIC-IV: Medical Information Mart for Intensive Care-IV
MIMIC-IV-CDM: Medical Information Mart for Intensive Care-IV-Clinical Decision Making
MT: machine translation
NLP: natural language processing
PICU: pediatric intensive care unit
PRISMA-ScR: Preferred Reporting Items for Systematic Reviews and Meta-Analyses extension for Scoping Reviews
PROBAST-AI: Prediction Model Risk of Bias Assessment Tool-Artificial Intelligence
RAG: retrieval-augmented generation
RoB: Risk of Bias
SOFA: Sequential Organ Failure Assessment
XGBoost: Extreme Gradient Boosting
