# Supplementary material for: Large Language Models in Critical Care Medicine: Scoping Review
Source: JMIR Med Inform. 2025 Nov 24;13:e76326. doi: 10.2196/76326 (PMC12778902; doi:10.2196/76326)
Supplement: Multimedia Appendix 1 [file medinform_v13i1e76326_app1.pdf]

Supplementary Materials of "Large Language Models in Critical Care Medicine: Scoping Review"

*Tongyue Shi, Jun Ma, Zihan Yu, Haowei Xu, Rongxin Yang, Minqi Xiong, Meirong Xiao, Yilin Li, Huiying Zhao, and Guilan Kong**

Note S1. Overview of LLMs in health and medicine. 2

Note S2. The process for selecting sources of evidence. 7

Note S3. Operational rules for PROBAST-AI ratings. 8

Note S4. Keyword co-occurrence network. 10

Table S1. Preferred Reporting Items for Systematic reviews and Meta-Analyses extension for Scoping Reviews (PRISMA-ScR) checklist. 11

Table S2. Search terms used across multiple databases for the scoping review. 14

Table S3. Design and performance summary for included studies. 15

Table S4. Risk of bias and applicability assessed using the PROBAST-AI. 23

Note S1. Overview of LLMs in health and medicine.

LLMs have transformed numerous fields through their unprecedented capabilities in understanding and generating natural language. Generally, LLMs refer to Transformer-based language models containing hundreds of billions or more parameters, trained on vast amounts of text data. Typical examples include GPT-3 [1], PaLM [2], and LLaMA [3].

The evolution of LLMs represents a complex and progressive journey intertwined with the advancements in generative models, sequence models, and pre-trained language models. Figure S1 shows the developing course of LLMs.

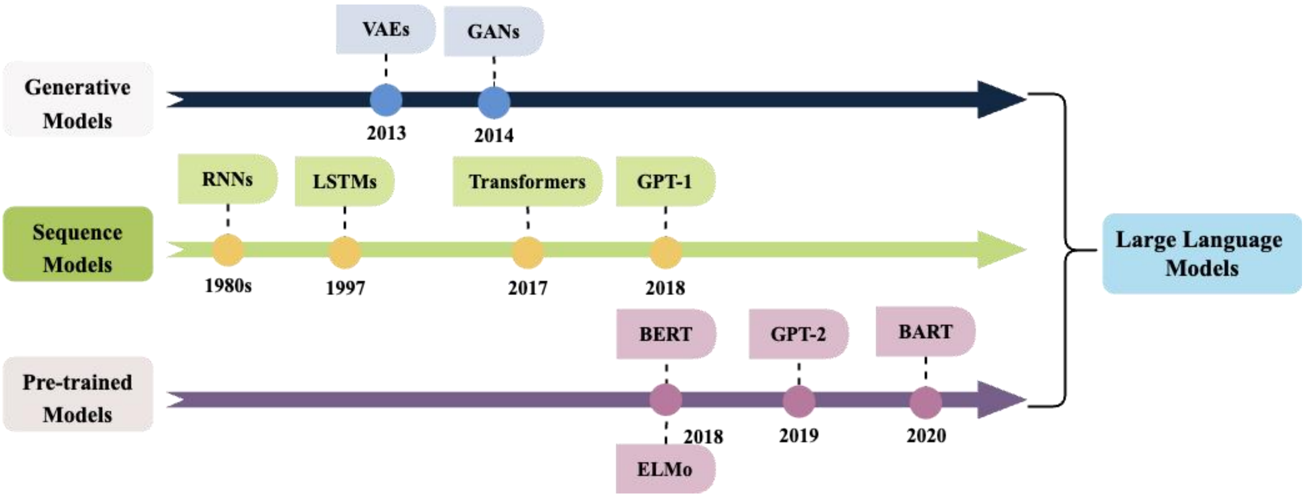

Figure S1. The developing course of large language models.

Generative Models Initially, research in this domain concentrated on generative models[4] for generating text [5], images [6], audio [7], and other AI-generated content [8] that closely resemble the training data. Generative adversarial networks (GANs) [9] and variational autoencoders (VAEs) [10] were typical generative models for text generation, machine translation, and image synthesis. These models aim to generate new examples with a similar distribution as the training set. However, their performance may be constrained by the availability of large-scale training datasets and the computational resources required for model training.

Sequence Models Sequence models operate on the principle that each word or token in a sequence can be predicted based on the preceding tokens [11]. The term "sequence" refers to the method of

processing and predicting data sequences. The Transformer architecture is an important milestone in the development of sequence models. It relies on a self-attention mechanism, allowing the model to weigh the importance of different words in each sequence to make predictions. Compared to recurrent neural networks (RNNs) [12] or long short-term memory networks (LSTMs) [13], Transformers can capture dependencies across the entire sequence more effectively, and they have become the backbone of many state-of-the-art LLMs.

Pre-trained Language Models As an early attempt, ELMo [14] captured context-aware word representations by pre-training a bidirectional LSTM network and subsequently fine-tuning it for specific downstream tasks. BERT [15] was developed based on the highly parallelizable Transformer architecture with self-attention mechanisms and pre-trained by a bidirectional language model on a large-scale unlabeled corpus. A "pre-training and fine-tuning" learning paradigm was developed in pre-trained language models (PLMs) and has inspired extensive follow-up research, which introduces different architectures such as GPT-2 [16] and BART [17]. This paradigm typically requires fine-tuning PLMs to adapt them to various downstream tasks.

Large Language Models Researchers have found that scaling PLMs can generally improve performance on downstream tasks following the scaling laws [18]. Some studies [1, 2] have explored the performance limits by training increasingly larger PLMs, such as GPT-3 with 175 billion parameters and PaLM with 540 billion parameters. These large-scale PLMs exhibit different behaviors from smaller PLMs like BERT with 330 million parameters and GPT-2 with 1.5 billion parameters. For instance, GPT-3 can address few-shot learning tasks through in-context learning, whereas GPT-2 performs poorly for similar tasks [1]. Consequently, the research community termed these large-scale PLMs as LLMs. As a typical application of LLMs, ChatGPT leverages the GPT series of LLMs for conversational purposes, demonstrating impressive human-like dialogue capabilities. Solving complex tasks which involve multiple steps is challenging for LLMs [19]. Nevertheless, prompting strategies such as chain-of-thought (CoT) [20], which include intermediate reasoning steps, can help tackle complex arithmetic, commonsense, and symbolic reasoning tasks.

LLMs exhibit substantial potential in different medical decision-making scenarios, including clinical decision support, medical document summarization, doctor-patient communication, and medical research. In clinical practice, LLMs can be utilized to provide supplemental treatments and diagnoses across different departments, such as internal medicine [21], surgery [22, 23], radiology [24, 25], and ophthalmology [26, 27]. The capabilities of summarizing and rephrasing information enable LLMs to generate detailed discharge summaries [28], radiology reports [29, 30], and other related medical documents, thereby reducing physicians' administrative burden. Furthermore, LLMs can automatize the international classification of diseases (ICD) coding process by extracting medical terms from clinical notes and assigning corresponding ICD codes, helping to improve coding efficiency and accuracy [31, 32]. The strong natural language understanding and generation capabilities of LLMs enable them to answer questions from patients with prostate cancer [33], nasal diseases [34], and liver cirrhosis [35], and can also provide emotional support to patients or caregivers [36]. ChatGPT has shown greater empathy than doctors when responding to patient inquiries [37]. In medical research, LLMs can serve as tools for literature retrieval and analysis [38], drug design and discovery [39], medical image segmentation [40], and medical language translation [41]. Utilizing LLMs for literature review and data analysis can help accelerate the research progress. Overall, applying LLMs in medicine can help improve clinical efficiency, support doctor-patient communication, and accelerate research progress to advance health and medicine.

References

1. Brown T, Mann B, Ryder N, Subbiah M, Kaplan JD, Dhariwal P, Neelakantan A, Shyam P, Sastry G, Askell A: **Language models are few-shot learners**. *Advances in neural information processing systems* 2020, **33**:1877-1901.
2. Chowdhery A, Narang S, Devlin J, Bosma M, Mishra G, Roberts A, Barham P, Chung HW, Sutton C, Gehrmann S: **Palm: Scaling language modeling with pathways**. *Journal of Machine Learning Research* 2023, **24**(240):1-113.
3. Han X, Zhang Z, Ding N, Gu Y, Liu X, Huo Y, Qiu J, Yao Y, Zhang A, Zhang L: **Pre-trained models: Past, present and future**. *AI Open* 2021, **2**:225-250.
4. Harshvardhan G, Gourisaria MK, Pandey M, Rautaray SS: **A comprehensive survey and analysis of generative models in machine learning**. *Computer Science Review* 2020, **38**:100285.
5. Hu Z, Yang Z, Liang X, Salakhutdinov R, Xing EP: **Toward controlled generation of text**. In: *International conference on machine learning: 2017*: PMLR; 2017: 1587-1596.

6. Sarkar K, Liu L, Golyanik V, Theobalt C: **Humangan: A generative model of human images**. In: *2021 International Conference on 3D Vision (3DV): 2021*: IEEE; 2021: 258-267.
7. Kim S, Lee S-G, Song J, Kim J, Yoon S: **FloWaveNet: A generative flow for raw audio**. *arXiv preprint arXiv:181102155* 2018.
8. Wu J, Gan W, Chen Z, Wan S, Lin H: **Ai-generated content (aigc): A survey**. *arXiv preprint arXiv:230406632* 2023.
9. Goodfellow I, Pouget-Abadie J, Mirza M, Xu B, Warde-Farley D, Ozair S, Courville A, Bengio Y: **Generative adversarial networks**. *Communications of the ACM* 2020, **63**(11):139-144.
10. Doersch C: **Tutorial on variational autoencoders**. *arXiv preprint arXiv:160605908* 2016.
11. Lipton ZC, Berkowitz J, Elkan C: **A critical review of recurrent neural networks for sequence learning**. *arXiv preprint arXiv:150600019* 2015.
12. Mikolov T, Karafiát M, Burget L, Cernocký J, Khudanpur S: **Recurrent neural network based language model**. In: *Interspeech: 2010*: Makuhari; 2010: 1045-1048.
13. Hochreiter S, Schmidhuber J: **Long short-term memory**. *Neural computation* 1997, **9**(8):1735-1780.
14. Peters ME, Neumann M, Iyyer M, Gardner M, Clark C, Lee K, Zettlemoyer L: **Deep contextualized word representations**.
15. Kenton JDM-WC, Toutanova LK: **Bert: Pre-training of deep bidirectional transformers for language understanding**. In: *Proceedings of naacL-HLT: 2019*; 2019: 2.
16. Radford A, Wu J, Child R, Luan D, Amodei D, Sutskever I: **Language models are unsupervised multitask learners**. *OpenAI blog* 2019, **1**(8):9.
17. Lewis M, Liu Y, Goyal N, Ghazvininejad M, Mohamed A, Levy O, Stoyanov V, Zettlemoyer L: **Bart: Denoising sequence-to-sequence pre-training for natural language generation, translation, and comprehension**. *arXiv preprint arXiv:191013461* 2019.
18. Kaplan J, McCandlish S, Henighan T, Brown TB, Chess B, Child R, Gray S, Radford A, Wu J, Amodei D: **Scaling laws for neural language models**. *arXiv preprint arXiv:200108361* 2020.
19. Wei J, Bosma M, Zhao VY, Guu K, Yu AW, Lester B, Du N, Dai AM, Le QV: **Finetuned language models are zero-shot learners**. *arXiv preprint arXiv:210901652* 2021.
20. Wei J, Wang X, Schuurmans D, Bosma M, Xia F, Chi E, Le QV, Zhou D: **Chain-of-thought prompting elicits reasoning in large language models**. *Advances in neural information processing systems* 2022, **35**:24824-24837.
21. Omiye JA, Gui H, Rezaei SJ, Zou J, Daneshjou R: **Large Language Models in Medicine: The Potentials and Pitfalls: A Narrative Review**. *Annals of Internal Medicine* 2024, **177**(2):210-220.
22. Puladi B, Gsaxner C, Kleesiek J, Hölzle F, Röhrig R, Egger J: **The impact and opportunities of large language models like ChatGPT in oral and maxillofacial surgery: a narrative review**. *International journal of oral and maxillofacial surgery* 2023.
23. Kothari A: **ChatGPT, large language models, and generative AI as future augments of surgical cancer care**. *Annals of Surgical Oncology* 2023, **30**(6):3174-3176.
24. D'Antonoli TA, Stanzione A, Bluethgen C, Vernuccio F, Ugga L, Klontzas ME, Cuocolo R, Cannella R, Koçak B: **Large language models in radiology: fundamentals, applications, ethical considerations, risks, and future directions**. *Diagnostic and Interventional Radiology* 2024, **30**(2):80.
25. Shen Y, Heacock L, Elias J, Hentel KD, Reig B, Shih G, Moy L: **ChatGPT and other large language models are double-edged swords**. In., vol. 307: Radiological Society of North America; 2023: e230163.

26. Hu X, Ran AR, Nguyen TX, Szeto S, Yam JC, Chan CK, Cheung CY: **What can Gpt-4 do for diagnosing rare eye diseases? A pilot study**. *Ophthalmology and Therapy* 2023, **12**(6):3395-3402.
27. Mihalache A, Popovic MM, Muni RH: **Performance of an artificial intelligence chatbot in ophthalmic knowledge assessment**. *JAMA ophthalmology* 2023, **141**(6):589-597.
28. Arora A, Arora A: **The promise of large language models in health care**. *The Lancet* 2023, **401**(10377):641.
29. Wu C, Zhang X, Zhang Y, Wang Y, Xie W: **Towards generalist foundation model for radiology**. *arXiv preprint arXiv:230802463* 2023.
30. Hyland SL, Bannur S, Bouzid K, Castro DC, Ranjit M, Schwaighofer A, Pérez-García F, Salvatelli V, Srivastav S, Thieme A: **MAIRA-1: A specialised large multimodal model for radiology report generation**. *arXiv preprint arXiv:231113668* 2023.
31. Yang Z, Batra SS, Stremmel J, Halperin E: **Surpassing GPT-4 Medical Coding with a Two-Stage Approach**. *arXiv preprint arXiv:231113735* 2023.
32. Liu J, Yang S, Peng T, Hu X, Zhu Q: **ChatICD: Prompt Learning for Few-shot ICD Coding through ChatGPT**. In: *2023 IEEE International Conference on Bioinformatics and Biomedicine (BIBM): 2023*: IEEE; 2023: 4360-4367.
33. Zhu L, Mou W, Chen R: **Can the ChatGPT and other large language models with internet-connected database solve the questions and concerns of patient with prostate cancer and help democratize medical knowledge?** *Journal of Translational Medicine* 2023, **21**(1):269.
34. Yoshiyasu Y, Wu F, Dhanda AK, Gorelik D, Takashima M, Ahmed OG: **GPT-4 accuracy and completeness against International Consensus Statement on Allergy and Rhinology: Rhinosinusitis**. In: *International Forum of Allergy & Rhinology: 2023*: Wiley Online Library; 2023: 2231-2234.
35. Yeo YH, Samaan JS, Ng WH, Ting P-S, Trivedi H, Vipani A, Ayoub W, Yang JD, Liran O, Spiegel B: **Assessing the performance of ChatGPT in answering questions regarding cirrhosis and hepatocellular carcinoma**. *Clinical and molecular hepatology* 2023, **29**(3):721.
36. Lokker C, Bagheri E, Abdelkader W, Parrish R, Afzal M, Navarro T, Cotoi C, Germini F, Linkins L, Haynes RB: **Deep learning to refine the identification of high-quality clinical research articles from the biomedical literature: Performance evaluation**. *Journal of Biomedical Informatics* 2023, **142**:104384.
37. Ayers JW, Poliak A, Dredze M, Leas EC, Zhu Z, Kelley JB, Faix DJ, Goodman AM, Longhurst CA, Hogarth M: **Comparing physician and artificial intelligence chatbot responses to patient questions posted to a public social media forum**. *JAMA internal medicine* 2023, **183**(6):589-596.
38. Qureshi R, Shaughnessy D, Gill KA, Robinson KA, Li T, Agai E: **Are ChatGPT and large language models "the answer" to bringing us closer to systematic review automation?** *Systematic Reviews* 2023, **12**(1):72.
39. Atas Guvenilir H, Doğan T: **How to approach machine learning-based prediction of drug/compound–target interactions**. *Journal of Cheminformatics* 2023, **15**(1):16.
40. Li Z, Li Y, Li Q, Wang P, Guo D, Lu L, Jin D, Zhang Y, Hong Q: **Lvit: language meets vision transformer in medical image segmentation**. *IEEE transactions on medical imaging* 2023.
41. Guo Y, Qiu W, Leroy G, Wang S, Cohen T: **Retrieval augmentation of large language models for lay language generation**. *Journal of Biomedical Informatics* 2024, **149**:104580.

Note S2. The process for selecting sources of evidence.

The literature screening involved a review of titles, abstracts, and keywords by two independent reviewers (T.S. and Z.Y.). This first step was designed to eliminate irrelevant articles based on the inclusion and exclusion criteria. Articles that passed this preliminary filter were subjected to a more detailed full-text review. The same reviewers thoroughly checked the full articles during this second phase to confirm their eligibility. Discrepancies between reviewers at any stage of the selection process were resolved through discussion. A third reviewer (G.K.) was consulted to make the final decision if a consensus could not be reached.

Note S3. Operational rules for PROBAST-AI ratings. (Three-level scale: Low (+), Unclear (?), High (–))

Risk of Bias (4 domains): (1) Participants: +: clearly defined ICU/HDU cohort; consecutive/near-consecutive sampling; pre-specified inclusion/exclusion; prediction time window defined. ?: sampling/time window not stated; mixed settings (eg, ICU+ED) without stratification. –: convenience or extreme case-control sampling causing spectrum bias; predominantly non-ICU data used for ICU tasks; large non-random exclusions without bias analysis. (2) Predictors: +: all inputs available before prediction time; consistent definitions/units; no leakage (no discharge summaries or post-outcome notes); feature/prompt/threshold selection confined to training folds. ?: predictor list or timing unclear; unclear whether LLM inputs mask outcome clues; selection/tuning procedure not described. –: use of post-outcome documents or outcome-revealing metadata; selection/tuning on full data or test sets; cross-fold/site leakage (same patient in train and test). (3) Outcome: +: recognized ICU standard with matching time window (eg, ICU mortality, ICU LOS, Sepsis-3); blinded ascertainment or objective algorithm; primary/secondary endpoints specified. ?: definition/measurement/timing not fully described; "clinical judgment" without standard. –: subjective, inconsistent endpoint; same source used as both input and reference; time window mismatch. (4) Analysis: +: robust internal validation (temporal split/k-fold/bootstrap), confidence intervals and calibration reported; missingness and class imbalance handled within training folds; one-time evaluation on the test set; external validation when available. ?: validation scheme or missing-data handling not stated; point estimates only, no CI/calibration. –: apparent performance only; tuning or thresholding on the test set; patient overlap between train/test; no calibration and no internal/external validation.

Applicability (3 domains): (1) Participants: +: matches target ICU/HDU population and resource level. ?: population insufficiently described. –: single-center highly specialized or non-ICU cohort misaligned with target use. (2) Predictors: +: variables/notes obtainable in real time in the target ICU without proprietary hardware or heavy manual annotation. ?: availability/latency unclear; definitions/interpretability unclear. –: dependencies on post-discharge data, proprietary devices, or intensive manual labeling not feasible for real-time ICU use. (3) Outcome: +: endpoint consistent

with target clinical question and local practice (eg, ICU mortality, 24-h AKI risk). ?: definition/measurement unclear. –: proxy or non-clinical endpoint (eg, readability only) not aligned with target use.

Overall rules: Overall RoB is typically High if any RoB domain is High; Unclear if no High but ≥1 Unclear; Low only if all RoB domains are Low (allowing lack of external validation when other criteria are rigorous). Overall Applicability is High if any applicability domain is High; Low if all are Low; otherwise Unclear. Missing data codes: NR (not reported), NA (not applicable).

Note S4. Keyword co-occurrence network.

As shown in Figure S2, 197 unique keywords were extracted from the literature using the VOSviewer software, among which "artificial intelligence" emerged as the most frequently occurring term, appearing in 17 articles with a total link strength of 145. This highlights the central role of AI in the reviewed studies. The keyword "chatgpt" also featured prominently, with 10 occurrences and a total link strength of 91, indicating a strong research interest in the capabilities or potentials of this specific model. Other notable keywords include "gpt-4," "large language model," "natural language processing" and "clinical decision support," each with unique role in the evolving landscape of LLMs. This underlines a significant gap in research that specifically targets the integration of LLMs within critical care settings.

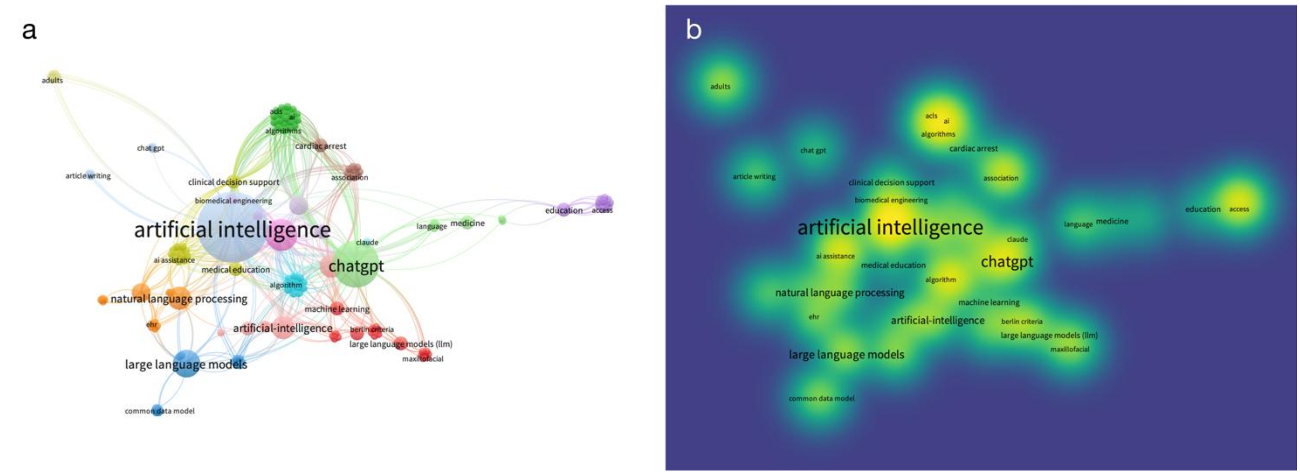

Figure S2. Keyword co-occurrence network analysis using the VOSviewer software. (a) Network Visualization, (b) Density Visualization.

Table S1. Preferred Reporting Items for Systematic reviews and Meta-Analyses extension for Scoping Reviews (PRISMA-ScR) Checklist.

SECTION	ITEM	PRISMA-ScR CHECKLIST ITEM	REPORTED ON PAGE #
**TITLE**			
Title	1	Identify the report as a scoping review.	1
**ABSTRACT**			
Structured summary	2	Provide a structured summary that includes (as applicable): background, objectives, eligibility criteria, sources of evidence, charting methods, results, and conclusions that relate to the review questions and objectives.	2
**INTRODUCTION**			
Rationale	3	Describe the rationale for the review in the context of what is already known. Explain why the review questions/objectives lend themselves to a scoping review approach.	3
Objectives	4	Provide an explicit statement of the questions and objectives being addressed with reference to their key elements (e.g., population or participants, concepts, and context) or other relevant key elements used to conceptualize the review questions and/or objectives.	3
**METHODS**			
Protocol and registration	5	Indicate whether a review protocol exists; state if and where it can be accessed (e.g., a Web address); and if available, provide registration information, including the registration number.	5
Eligibility criteria	6	Specify characteristics of the sources of evidence used as eligibility criteria (e.g., years considered, language, and publication status), and provide a rationale.	5
Information sources*	7	Describe all information sources in the search (e.g., databases with dates of coverage and contact with authors to identify additional sources), as well as the date the most recent search was executed.	5
Search	8	Present the full electronic search strategy for at least 1 database, including any limits used, such that it could be repeated.	5
Selection of sources of evidence†	9	State the process for selecting sources of evidence (i.e., screening and eligibility) included in the scoping review.	5
Data charting process‡	10	Describe the methods of charting data from the included sources of evidence (e.g., calibrated forms or forms that have been tested by the team before their use, and whether data charting was done independently or in duplicate) and any processes for obtaining and	5

SECTION	ITEM	PRISMA-ScR CHECKLIST ITEM	REPORTED ON PAGE #
		confirming data from investigators.	
Data items	11	List and define all variables for which data were sought and any assumptions and simplifications made.	5
Critical appraisal of individual sources of evidence§	12	If done, provide a rationale for conducting a critical appraisal of included sources of evidence; describe the methods used and how this information was used in any data synthesis (if appropriate).	5
Synthesis of results	13	Describe the methods of handling and summarizing the data that were charted.	6
**RESULTS**			
Selection of sources of evidence	14	Give numbers of sources of evidence screened, assessed for eligibility, and included in the review, with reasons for exclusions at each stage, ideally using a flow diagram.	7
Characteristics of sources of evidence	15	For each source of evidence, present characteristics for which data were charted and provide the citations.	12-20
Critical appraisal within sources of evidence	16	If done, present data on critical appraisal of included sources of evidence (see item 12).	12-20
Results of individual sources of evidence	17	For each included source of evidence, present the relevant data that were charted that relate to the review questions and objectives.	12-20
Synthesis of results	18	Summarize and/or present the charting results as they relate to the review questions and objectives.	12-20
**DISCUSSION**			
Summary of evidence	19	Summarize the main results (including an overview of concepts, themes, and types of evidence available), link to the review questions and objectives, and consider the relevance to key groups.	20
Limitations	20	Discuss the limitations of the scoping review process.	28
Conclusions	21	Provide a general interpretation of the results with respect to the review questions and objectives, as well as potential implications and/or next steps.	28-29
**FUNDING**			
Funding	22	Describe sources of funding for the included sources of evidence, as well as sources of funding for the scoping review. Describe the role of the funders of the scoping review.	29

JBI = Joanna Briggs Institute; PRISMA-ScR = Preferred Reporting Items for Systematic reviews and Meta-Analyses extension for Scoping Reviews.

* Where *sources of evidence* (see second footnote) are compiled from, such as bibliographic databases, social media platforms, and Web sites.

† A more inclusive/heterogeneous term used to account for the different types of evidence or data sources (e.g., quantitative and/or qualitative research, expert opinion, and policy documents) that may be eligible in a scoping review as opposed to only studies. This is not to be confused with *information sources* (see first footnote).

‡ The frameworks by Arksey and O’Malley (6) and Levac and colleagues (7) and the JBI guidance (4, 5) refer to the process of data extraction in a scoping review as data charting.

§ The process of systematically examining research evidence to assess its validity, results, and relevance before using it to inform a decision. This term is used for items 12 and 19 instead of "risk of bias" (which is more applicable to systematic reviews of interventions) to include and acknowledge the various sources of evidence that may be used in a scoping review (e.g., quantitative and/or qualitative research, expert opinion, and policy document).

From: Tricco AC, Lillie E, Zarin W, O'Brien KK, Colquhoun H, Levac D, et al. PRISMA Extension for Scoping Reviews (PRISMA-ScR): Checklist and Explanation. Ann Intern Med. 2018;169:467–473. doi: 10.7326/M18-0850.

Table S2. Search terms used across multiple databases for the scoping review.

Database	Search Terms	Number of Records Found
PubMed	(“Critical Care” [MeSH] OR “Intensive Care Units”[MeSH] OR “Critical Illness”[MeSH] OR “Critical Care”[TIAB] OR “Intensive Care”[TIAB] OR “Critical Illness”[TIAB] OR “ICU”[TIAB]) AND (“Large Language Model”[TIAB] OR “LLM”[TIAB] OR “Generative Pre-trained Transformer”[TIAB] OR “GPT”[TIAB] OR “Generative Artificial Intelligence”[TIAB] OR “Generative AI”[TIAB])	145
Scopus	TITLE-ABS-KEY (“Critical Care” OR “Intensive Care Units” OR “Critical Illness” OR “Intensive Care” OR “ICU”) AND TITLE-ABS-KEY (“Large Language Model” OR “LLM” OR “Generative Pre-trained Transformer” OR “GPT” OR “Generative Artificial Intelligence” OR “Generative AI”)	235
Web of Science	TS=(“Critical Care” OR “Intensive Care Units” OR “Critical Illness” OR “Intensive Care” OR “ICU”) AND TS=(“Large Language Model” OR “LLM” OR “Generative Pre-trained Transformer” OR “GPT” OR “Generative Artificial Intelligence” OR “Generative AI”)	396
Embase	(‘Critical Care’/exp OR ‘Intensive Care Units’/exp OR ‘Critical Illness’/exp OR ‘Intensive Care’/exp OR ‘ICU’/exp OR ‘Critical Care’ OR ‘Critical Illness’ OR ‘Intensive Care’ OR ‘ICU’) AND (‘Large Language Model’ OR ‘LLM’ OR ‘Generative Pre-trained Transformer’ OR ‘GPT’ OR ‘Generative Artificial Intelligence’ OR ‘Generative AI’)	1,032
CINAHL	(MH “Critical Care” OR MH “Intensive Care Units” OR MH “Critical Illness” OR TI “Critical Care” OR TI “Intensive Care” OR TI “Critical Illness” OR TI “ICU” OR AB “Critical Care” OR AB “Intensive Care” OR AB “Critical Illness” OR AB “ICU”) AND (TI “Large Language Model” OR AB “Large Language Model” OR TI “LLM” OR AB “LLM” OR TI “Generative Pre-trained Transformer” OR AB “Generative Pre-trained Transformer” OR TI “GPT” OR AB “GPT” OR TI “Generative Artificial Intelligence” OR AB “Generative Artificial Intelligence” OR TI “Generative AI” OR AB “Generative AI”)	23
IEEE Xplore	(‘Critical Care’ OR ‘Intensive Care Units’ OR ‘Critical Illness’ OR ‘Intensive Care’ OR ‘ICU’) AND (‘Large Language Model’ OR ‘LLM’ OR ‘Generative Pre-trained Transformer’ OR ‘GPT’ OR ‘Generative Artificial Intelligence’ OR ‘Generative AI’)	237
ACM Digital Library	[[All: “critical care”] OR [All: “intensive care unit”] OR [All: “critical illness”] OR [All: “intensive care”] OR [All: “icu”]] AND [[All: “large language model”] OR [All: “llm”] OR [All: “generative pre-trained transformer”] OR [All: “gpt”] OR [All: “generative artificial intelligence”] OR [All: “generative ai”]]	274

Table S3. Design and performance summary for included studies.

Study	Metrics (AUROC, AUPRC, and F1)	Setting	Implementation effect on patient outcomes	Validation design	External validation (environment)
Savage et al.	AUPRC: 0.82 (95% CI 0.75–0.89); AUROC: 0.89 (95% CI 0.84–0.94); sensitivity: 67%; specificity: 95%	ICU	Reduced unnecessary alerts by 20%, increased positive alert applicability from 76% to 90.8%	Training on 500 notes; development (n=300) and test (n=300) sets; retrospective evaluation of classification performance	Not reported
Levin et al.	AUROC/AUPRC/F1; Claude-2 significantly outperformed ChatGPT-4 in accuracy and response time (statistical significance implied)	Neonatal ICU	Indicates LLMs' emerging capacity in neonatal decision support, but still inferior to clinical experts; emphasizes need for refinement	Cross-sectional comparative study; models and nurses presented with same written scenarios; responses evaluated by neonatal nurse practitioners for accuracy, completeness, and latency	Not reported
Pham et al.	Median step-level accuracy: 85% (cardiac arrest), 30% (bradycardia); median simulation-level accuracy across 20 attempts: 69% (cardiac arrest), 42% (bradycardia)	ICU	Indicates potential but inconsistent decision support; risks of omission, repetition, and misinformation highlight need for oversight	Prospective exploratory simulation with 2 scenarios, each repeated 20 times; correctness scored stepwise	Not reported
Huespe et al.	GPT-3.5 scored higher on Likert (median 4.5 vs 3.82 and 3.6; p<0.001); low ability to distinguish AI vs human (sensitivity 22.4%, specificity 57.6%)	ICU	Demonstrates GPT-3.5's capacity to generate high-quality academic prose; indirect educational or research efficiency benefits	Blinded survey with 80 researchers rating background sections on Likert scales; source (human/AI) detection task included	Not reported
Si et al.	F1-measures across four corpora: 90.25 (i2b2 2010), 93.18 (partial match, i2b2	ICU	Indirect: improved concept extraction accuracy supports downstream clinical	Comparative modeling: tested multiple embedding techniques on established corpora;	Not reported

	2012), 80.74 (SemEval 2014), 81.65 (SemEval 2015)		NLP tasks, which may enhance decision support and research quality	analyzed effect of domain-specific pretraining and pretraining duration	
Almazyad et al.	Not applicable	Pediatric ICU	ChatGPT-4 improved clarity of complex pediatric end-of-life case scenarios and supported thematic summarization of expert panel discussions, but no patient-level outcomes assessed.	Qualitative descriptive study during a pediatric palliative critical-care conference; ChatGPT-4 revised two case scenarios and generated summaries from panel transcripts.	Not reported
Chung et al.	AUROC/AUPRC/F1; ChatGPT-4 preferred in 60% factual ($p<0.001$), specialist preferred in 67% reasoning ($p<0.0001$); completeness higher for ChatGPT-4 ($p=0.02$)	ICU	ChatGPT-4 may enhance factual teaching but lacks adequacy in clinical reasoning; highlights role in education not direct patient care	Comparative paired evaluation of 198 response pairs by 10 PICU specialists; assessed preference, completeness, accuracy, integration	Not reported
Abdullahi et al.	Not applicable	ICU	By lowering technical barriers, clinicians can more easily access and analyze ICU data, potentially accelerating research and improving care through data-driven insights	Descriptive development and functionality demonstration; user interface and operational flow validated through prototype stages	Not reported
Benboujja et al.	Performance in capturing predefined key clinical events: GPT-4 API: 41.5 ± 15.2%; ChatGPT: 19.2 ± 20.9%; Llama 2: 16.5 ± 14.1% ($p = 0.002$)	ICU	Demonstrates LLM potential to streamline documentation and reduce clinician workload, though moderate accuracy and omissions suggest need for caution	Pilot feasibility study: prompting structure developed on 5 cases, tested on 6 ICU episodes; summaries evaluated by intensivists on event capture, readability, organization, succinctness, hallucinations	Not reported

Lu et al.	Accuracy: GPT-4o: 93.3%; Mistral: 87.9%; Llama 3.1: 87.5%; GPT-4o-mini: 83.0%; GPT-3.5-turbo: 72.7%; human physicians: ~61.9%	ICU	Highlights LLMs' superior knowledge recall which may inform educational or decision-support tools; no direct patient outcome assessed	Retrospective benchmarking against human performance on standardized question bank	Not reported
Madden et al.	Not applicable	ICU	May reduce clinician documentation burden and enhance information retrieval; caution needed due to hallucination risk	Descriptive/observational evaluation of LLM-generated summaries, assessed for conciseness and utility	Not reported
Liu et al.	AUC: 0.821 (95% CI: 0.698–0.943) for GPT-4.0; precision: 76.1%, specificity: 78.6%, sensitivity: 75.4% vs specialist physicians precision: 80.3%, specificity: 71.4%, sensitivity: 82.5%	ICU	Demonstrates GPT-4.0's diagnostic accuracy comparable to specialists; potential to assist timely intubation decisions in resource-limited or support settings	Prospective multicenter cohort study (n = 71); participants (GPT-3.5, GPT-4.0, specialists, non-specialists) answered six-alternative forced-choice questionnaires; ROC analyses used for evaluation	Not reported
Nolan et al.	Macro F1-score: 0.84 (doubt markers), 0.79 (stigmatizing labels); precision & recall ranged from 0.71–0.86; accuracy ~0.87, comparable to human annotator agreement	ICU	Enables detection of biased or stigmatizing language; may support interventions to improve provider communication and reduce documentation-driven bias	Lexicon development from literature and embedding models; annotation of 1000 sentence samples; supervised classifier training and evaluation; performance compared to human annotation	Not reported
Shah-Mohammadi et al.	F1: ~0.92 (substance use detection)	ICU-related	Not directly evaluated	Retrospective text data evaluation	Not reported
Nawab et al.	F1: 0.82–0.89 (depending on ICD granularity)	ICU-related	Not directly evaluated	Retrospective evaluation on clinical documentation	Not reported
Soleimani et al.	F1: 0.85–0.88 (key findings extraction)	ICU-related	Not directly evaluated	Prospective vs retrospective evaluation of generated reports	Not reported
Oh et al.	AUROC: 0.78–0.81;	ICU	Not directly	Retrospective cohort,	Korean

	AUPRC: 0.35–0.40		implemented in clinical care; predictive only	in-context learning	multicenter ICU database (external validation across centers)
Urquhart et al.	F1: 0.88–0.91	ICU	Not directly implemented	Retrospective evaluation on ICU notes	Irish multicenter ICU dataset
Pabon et al.	Not applicable	Cardiovascular ICU	Provides prognostic data; clinical effect on outcomes described but not via ML implementation	Prospective randomized clinical trial	Multinational DELIVER trial sites
Akhondi-Asl et al.	Not applicable	Pediatric ICU	Not directly implemented; evaluated potential as clinical decision support	Retrospective cohort study; 130 admission notes randomly selected, model outputs rated by 5 critical care experts	Not reported
Liu et al.	Not applicable	ICU	No direct patient outcome data; potential indirect effect via improving simulation education quality, teaching efficiency, and learner engagement	Semi-structured interviews (n=13), thematic analysis, guided by Technology Acceptance Model, double-coding with 85% agreement, SRQR reporting standards	Not reported
Berger et al.	Not applicable	ICU	Identified potential risks of miscommunication that could compromise patient safety; recommendations emphasize reducing ambiguity to improve outcomes	Qualitative design, interviews and thematic analysis of ICU staff experiences and perceptions	Not reported
Kurz et al.	Accuracy: open-source VLMs up to 40.4%, GPT-4o at 68.1%	Emergency and ICU	Indicates potential for GPT-4o to improve diagnostic accuracy in acute care; open models currently insufficient, implying	Multimodal diagnostic benchmark with standardized dataset and direct comparison across models	Not reported

			limited impact on patient outcomes without further development		
Pham et al.	AUROC: 0.796; Sensitivity: 75%; Specificity: 84.2% (no AUPRC or F1 provided)	Neonatal ICU	Demonstrated moderate diagnostic accuracy, suggesting ChatGPT-4o could assist early detection of PV-IVH and potentially improve timely intervention in underserved settings	Cross-sectional diagnostic study; 35 infants, comparisons with radiologists; outcome metrics and inter-rater agreement (Kappa = 0.595) assessed	Not reported
Shi et al.	C-index: SWEDEHEART-AI 0.72; Qwen-2 0.65; Llama-3 0.56. AUROC consistent over time; calibration overestimation (O/E ratio 0.478); no AUPRC/F1 reported	ICU	SWEDEHEART-AI shows better risk stratification performance; potential to enhance early intervention decisions, LLMs show moderate potential but need further tuning	`Retrospective cohort modeling: derivation via SWEDEHEART registry trained ANN, LLMs applied to discharge text; evaluation via C-index, time-dependent AUROC, calibration plots, Kaplan-Meier, decision curve analysis	Not reported
Yitzhaki et al.	AUROC/AUPRC/F1; preference in factual knowledge 60% for ChatGPT-4; specialist preferred in clinical reasoning 67%; completeness advantage (p=0.02) in factual; lower accuracy in reasoning (p<0.0001)	PICU	Indicates ChatGPT-4 may enhance factual knowledge dissemination and completeness, but lacks sufficient clinical reasoning for standalone use, impacts educational support rather than direct patient outcomes	Comparative paired evaluation: experts rated 198 paired responses covering factual and clinical reasoning; metrics included preference, completeness, accuracy, integration potential	Not reported
Williams et al.	AUROC/AUPRC/F1; outcomes categorized as correct/partially correct/incorrect/other; differences significant (P=0.007)	ICU	Indicates AI potential but inconsistent accuracy; reliance could endanger patient safety without refinement	Comparative cross-sectional analysis of 43 responses per model; categorized and statistically compared	Not reported

Yang et al.	Not applicable	ICU	Indirect: by enabling easier access to critical care data, it may accelerate research and detection of patterns that could inform interventions	Descriptive development and usability demonstration; technical validation in deploying and querying databases via UI and LLM	Not reported
Workum et al.	AUROC/AUPRC/F1; report accuracy: GPT-4o: 93.3%, Llama 3.1 70B: 87.5%, Mistral: 87.9%, GPT-4o-mini: 83.0%, GPT-3.5-turbo: 72.7%; human physicians' practice test accuracy ~61.9%	ICU	Demonstrated superior knowledge acquisition potential of LLMs vs human trainees; indirect implications for decision support but no direct patient outcome data	Retrospective benchmarking with 1,181 MCQs plus 77-MCQ practice test by physicians; metrics included accuracy and consistency; cost/energy consumption also analyzed	Not reported
Yang et al.	AUROC/AUPRC/F1; Main metric: Accuracy (GPT-4: 71.6%); Variance reported; CoT prompting improved accuracy to 77.4%	ICU	Educational impact: suggests improved learning support via enhanced accuracy; no direct patient outcomes	Comparative experimental design: each model queried five times; metrics included accuracy, variance, comparison to student performance, and effect of prompting strategies	Not reported
Ding et al.	Up to 4.48% improvement in accuracy vs baseline; reported improvement in AUROC, AUPRC, F1 for heart failure prediction (exact values not given in summary)	ICU	Indirect; improved predictive performance may enable earlier detection and intervention, potentially improving patient outcomes	Retrospective predictive modeling with train/validation/test split; compared to baseline models; ablation and feature importance analyses conducted	Not reported
Walker et al.	Macro F1: 0.84 (doubt markers), 0.79 (stigmatizing labels); precision & recall 0.71–0.86; accuracy ~0.87 (comparable to	ICU	Indirect; by identifying linguistic bias, the tool enables future interventions for reducing stigma, which may improve	Lexicon development (literature + embedding + human refinement), annotation of sampled sentences, supervised classifier training and	Not reported

	human annotator agreement)		patient-provider communication and equity in care	evaluation , reported alignment with human labeling accuracy	
Chen et al.	Not applicable	ICU	Indirect: enhancing accessibility of high-quality critical care education across language barriers may support global training; potential downstream benefit to patient care	Comparative evaluation with professional human translation as reference; used blinded bilingual clinician Likert scoring, BLEU automated scoring, and usability surveys	Not reported
Ucdal et al.	AUC: e.g., severity classification ~0.886–0.892, drainage decision AUC ~0.874, nutrition timing AUPRC ~0.856; F1 overall ~0.835; other metrics: accuracy 85% (mild), 82% (severe); precision, recall included	ICU	Direct: AI-driven recommendations align with guidelines and scoring systems, offering consistent decision support that could improve care quality where specialist access is limited	Retrospective simulation study on 512 cases; compared AI recommendations to guideline criteria and scoring systems; performance measured via AUC, AUPRC, precision, recall, F1	Not reported
Balta et al.	Not applicable	ICU	Highlights risk of hallucination despite improved appropriateness; inconsistent outputs limit reliable clinical impact	Cross-sectional comparison: 50 standardized clinical questions evaluated independently by 2 intensivists	Not reported
Zhu et al.	AUROC between 0.77–0.89; combining text with clinical variables improved predictive accuracy; AUPRC/F1 not specified	ICU	Potential to support timely AKI recognition and intervention; suggests multimodal integration benefits outcomes	Comparative modeling with six CNN-based approaches; evaluated use of embedding types and inclusion of clinical variables	Not reported
Pathak et al.	F1-scores: 74.5% (Emory dataset), 64.22% (Grady dataset)	ICU	Automates ARDS detection from radiology reports; potential to support	Multi-site retrospective validation using real radiology notes, with traditional NLP pipeline	Demonstrated external validity across two

			early recognition and intervention	and performance metrics	independent hospital datasets
Liu et al.	Not applicable	ICU	By improving mortality prediction from text, can support earlier risk stratification and intervention, LLMs currently insufficient for reliable predictive tasks	Retrospective modeling with development and comparison across multiple models; LLMs evaluated under varied prompting environments	Not reported
Turan et al.	Parameter-level accuracy: pH, oxygenation, sodium, chloride: 100%; hemoglobin: 92.5%; bilirubin: 72.5%	ICU	Potential to expedite ABG interpretation and support decision-making; needs oversight in complex metabolic interpretations	Prospective observational study; blind comparison to two expert anesthesiologists on 400 samples	Not reported
Wang et al.	Success rate: 73.5%; fundamental knowledge accuracy: 81.94%; single-choice: 76.72%; multiple-choice: 51.32% ($p < 0.001$)	ICU	Suggests potential utility in decision support and medical education; indirect impact on patient care with need for supervision	Cross-sectional analysis based on 600 exam questions, expert-scored	Not reported
Yang et al.	F1-score: 76.76 (sepsis dataset); 65.42 (CMeEE few-shot); compared to Qwen2 (43.77) and Llama3 (48.39)	ICU	Indirect: enhances structural understanding of sepsis, facilitating research and decision support systems	Retrospective knowledge graph construction with entity/relation extraction and comparative model evaluation	Not reported

The order and cite of the articles is the same as Table 1 in the main text.

Table S4. Risk of bias and applicability assessed using the PROBAST-AI.

Study	Risk of Bias				Applicability			Overall	
	1. Participants	2. Predictors	3. Outcome	4. Analysis	1. Participants	2. Predictors	3. Outcome	Risk of Bias	Applicability
Savage et al.	+	+	+	?	+	+	+	?	+
Levin et al.	?	?	+	?	?	?	+	?	?
Pham et al.	–	–	?	?	–	–	–	–	–
Huespe et al.	–	–	–	–	–	–	–	–	–
Si et al.	?	+	–	+	?	–	–	?	–
Almazyad et al.	–	–	–	–	–	–	–	–	–
Chung et al.	?	?	+	?	?	?	+	?	?
Abdullahi et al.	–	–	–	–	–	–	–	–	–
Benboujja et al.	–	–	–	–	–	–	–	–	–
Lu et al.	–	–	–	–	–	–	–	–	–
Madden et al.	+	?	–	?	+	?	–	–	–
Liu et al.	?	?	+	?	?	?	+	?	?
Nolan et al.	–	–	–	–	–	–	–	–	–
Shah-Mohammadi et al.	?	+	–	+	?	–	–	–	–
Nawab et al.	?	+	–	+	?	–	–	–	–
Soleimani et al.	?	?	–	?	?	–	–	–	–
Oh et al.	+	?	+	?	+	+	+	?	+
Urquhart et al.	?	?	–	?	?	?	–	–	–
Pabon et al.	?	?	+	?	–	?	+	?	–
Akhondi-Asl et al.	+	+	?	+	+	+	?	?	?

Liu et al.	–	–	–	–	–	–	–	–	–
Berger et al.	–	–	–	–	–	–	–	–	–
Kurz et al.	?	?	?	+	–	?	–	?	–
Pham et al.	?	?	+	?	?	?	+	?	?
Shi et al.	+	?	+	?	+	+	+	?	+
Yitzhaki et al.	?	–	?	?	–	–	–	–	–
Williams et al.	–	–	?	?	–	–	–	–	–
Yang et al.	–	–	–	–	–	–	–	–	–
Workum et al.	–	–	?	+	–	–	–	–	–
Yang et al.	–	–	?	+	–	–	–	–	–
Ding et al.	?	?	?	?	?	?	?	?	?
Walker et al.	?	+	–	+	?	–	–	–	–
Chen et al.	–	–	–	+	–	–	–	–	–
Ucdal et al.	?	–	–	?	?	–	–	–	–
Balta et al.	–	–	?	+	–	–	–	?	–
Zhu et al.	+	?	+	?	+	+	+	?	+
Pathak et al.	?	+	+	+	+	+	+	+	+
Liu et al.	+	+	+	+	+	+	+	+	+
Turan et al.	?	–	+	?	?	–	–	–	–
Wang et al.	–	–	–	+	–	–	–	–	–
Yang et al.	+	?	–	+	?	–	–	–	–

The plus symbol (+) indicates a low risk of bias (ROB) or low concern for applicability; the minus symbol (−) means high ROB or high concern for applicability; the question mark (?) implies unclear ROB or unclear concern for applicability. The order and cite of the articles is the same as Table 1 in the main text.
